# Supplementary material for: The effect of life course socioeconomic position on crystallised cognitive ability in two large UK cohort studies: a structured modelling approach
Source: BMJ Open. 2017 Jun 2;7(5):e014461. doi: 10.1136/bmjopen-2016-014461 (PMC5541359; doi:10.1136/bmjopen-2016-014461)
Supplement: Supplementary data [file bmjopen-2016-014461supp001.pdf]

Appendix 1

Multiple imputation was carried out using the Stata command *ice*, created by Patrick Royston (118;119), which samples from the posterior predictive distribution $p(Y_{missing} | Y_{observed}, X)$. For 20 imputations, 20 independent selections are made from the posterior predictive distribution. All the variables which are thought to predict or be associated with the missing values should be included in the model used to form the posterior predictive distribution, the imputation model. When choosing which variables to include in the imputation model, it is important to also include any variables that may be important in subsequent analyses, including the variable(s) with missing data and the outcome of interest. It has been shown that including as many explanatory variables in the imputation model as possible makes the MAR assumption more plausible (van Buuren et al., 1999). Although including redundant predictors may be expected to reduce the precision of the final estimates, Kenward and Carpenter (Kenward and Carpenter, 2007) note that this effect is typically not large, concluding that it is better to err on the side of including too many variables in the imputation model rather than too few, as excluding an important predictor of missingness could cause bias.

van Buuren (van Buuren et al., 1999) and Carpenter and Plewis (Carpenter and Plewis, 2011) suggest similar methods for selecting the variables to include in the imputation model from a large database:

1. Include all variables that appear in the complete-data model of interest.
2. Include the variables which were predictive of missing data.
3. Check whether the variables included in step 2 were associated with the variables in the model of interest.
4. Auxiliary variables can also be included to make the assumption of MAR more likely. Auxiliary variables are predictive of any of the variables in the imputation model.

Binary variables were imputed using logistic regression, ordered categorical variables using ordered logistic regression, and unordered categorical variables using multinomial regression.

Variables included in the imputation model for Whitehall II men, in addition to those in the model of interest

Phase of data collection	Variables
1	Year of birth, longstanding illness, current cigarette smoker, age when finished full time education, isolation scale score, usually pressed for time, believe no one cares much about you, state of health in the last year, accommodation type, mother's age when finished education
3	Job involves travel away from home, marital status, last recorded civil service grade, memory score, AH4 score, verbal fluency (S-words, animals), Mill Hill score
4	Ever told had anxiety, ever told had depression,
5	Childhood emotional deprivation, childhood material deprivation, ever told high blood pressure, deprivation score, feel might as well give up, financial security in the next 10 years, last recorded civil service grade, memory score, AH4 score, verbal fluency (S-words, animals), Mill Hill score
7	Clinic or home visit, still working as a civil servant, general health, CASP total score, my health stops me doing things I want to do, MMSE total score, memory score, AH4 score, verbal fluency (S-words, animals), Mill Hill score
9	General health, difficulty paying bills, MMSE total score, marital status, last recorded civil service grade, memory score, AH4 score, verbal fluency (S-words, animals)

Variables included in the imputation model for Whitehall II women, in addition to those in the model of interest

Phase of data collection	Variables
1	Year of birth, longstanding illness, current cigarette smoker, age when finished full time education, isolation scale score, usually pressed for time, state of health in the last year, accommodation type, mother's age when finished education, satisfied by job as a whole, civil service grade
3	Job involves travel away from home, marital status, last recorded civil service grade, memory score, AH4 score, verbal fluency (S-words, animals), Mill Hill score
4	Ever told had depression
5	Childhood emotional deprivation, childhood material deprivation, ever told high blood pressure, feel might as well give up, last recorded civil service grade, memory score, AH4 score, verbal fluency (S-words, animals), Mill Hill score
7	Clinic or home visit, still working as a civil servant, general health, my health stops me doing things I want to do, MMSE total score, memory score, AH4 score, verbal fluency (S-words, animals), Mill Hill score
9	General health, difficulty paying bills, MMSE total score, marital status, last recorded civil service grade, memory score, AH4 score, verbal fluency (S-words, animals)

Variables included in the imputation model for NSHD men, in addition to those in the model of interest

Participant's Age	Variables
0	Father's occupational SEP
2	Childhood household amenities
4	Child has own bed, clothes repair, yard or garden, child's shoes, cleanliness of house, cleanliness of child, mother's management and understanding of the child, crowding, repair of dwelling
8	Reading score, sentence completion score, picture intelligence score, vocabulary score
10	Difficult to discipline
11	Father's occupational SEP, non-verbal reasoning score, reading score, verbal reasoning score, arithmetic score, vocabulary score, household amenities
13	Have to stick up for myself
15	Non-verbal reasoning score, reading score, verbal reasoning score, mathematics score, truancy during the past year
26	Own occupational SEP, happiness, job stress, brought up in a faith, currently have a religion
36	Marital status, current smoking status, ever been unemployed, own occupational SEP, ever had cancer, does religious upbringing effect life now
43	How many friends or relatives could you visit at any time without waiting for an invite, verbal memory score
53	Verbal fluency score

Variables included in the imputation model for NSHD women, in addition to those in the model of interest

Participant's Age	Variables
0	Father's occupational SEP
2	Childhood household amenities
4	Child has own bed, clothes repair, yard or garden, child's shoes, cleanliness of house, cleanliness of child, mother's management and understanding of the child, crowding, repair of dwelling, age of dwelling
8	Reading score, sentence completion score, picture intelligence score, vocabulary score
10	Difficult to discipline
11	Father's occupational SEP, non-verbal reasoning score, reading score, verbal reasoning score, arithmetic score, vocabulary score, household amenities
13	Have to stick up for myself
15	Non-verbal reasoning score, reading score, verbal reasoning score, mathematics score, truancy during the past year
26	Own occupational SEP, happiness, job stress, brought up in a faith, currently have a religion
31	Hospital admissions
36	Marital status, current smoking status, ever been unemployed, own occupational SEP, ever had cancer, does religious upbringing effect life now, blood pressure, how confident feel in myself
43	Head of household SEP, verbal memory score
53	Verbal fluency score

References:

CARPENTER, J. & PLEWIS, I. (2011) Analysing Longitudinal Studies with Non-response: Issues and statistical methods. IN WILLIANS, M. & VOGT, W. (Eds.) *The SAGE Handbook of Innovation in Social Research Methods.* SAGE Publications Ltd.

KENWARD, M. G. & CARPENTER, J. (2007) Multiple imputation: current perspectives. *Statistical Methods in Medical Research,* 16**,** 199-218.

VAN BUUREN, S., BOSHUIZEN, H. C. & KNOOK, D. L. (1999) Multiple imputation of missing blood pressure covariates in survival analysis. *Statistics in medicine,* 18**,** 681-694.
