# Supplementary material for: The effect of life course socioeconomic position on crystallised cognitive ability in two large UK cohort studies: a structured modelling approach
Source: BMJ Open. 2017 Jun 2;7(5):e014461. doi: 10.1136/bmjopen-2016-014461 (PMC5541359; doi:10.1136/bmjopen-2016-014461)
Supplement: Supplementary data [file bmjopen-2016-014461supp002.pdf]

Supplementary Table 1: Complete case results of tests comparing alternative life course hypotheses for crystallised cognitive ability (NSHD: NART score, Whitehall II: Mill Hill test score) with the saturated model (NSHD models are unadjusted, Whitehall II models are adjusted for age and number of times the Mill Hill test has previously been taken.

	NSHD				Whitehall II			
	Women		Men		Women		Men	
**Hypothesis**	F statistic	P-value*	F statistic	P-value*	F statistic	P-value*	F statistic	P-value*
No effect	80.72	<0.0001	53.99	<0.0001	31.16	<0.0001	70.33	<0.0001
Accumulation models								
Accumulation	4.90	0.0001	4.37	0.0002	4.74	0.0001	12.32	<0.0001
Adult accumulation	7.18	<0.0001	3.24	0.0037	1.85	**0.0861**	5.73	<0.0001
Sensitive period	2.02	**0.0891**	0.69	**0.5967**	0.93	**0.4459**	2.80	0.0248
Critical period models								
Childhood	51.80	<0.0001	45.41	<0.0001	28.49	<0.0001	74.81	<0.0001
Early adulthood	23.43	<0.0001	21.63	<0.0001	13.89	<0.0001	40.90	<0.0001
Adulthood	52.01	<0.0001	21.11	<0.0001	8.55	<0.0001	19.16	<0.0001
Social mobility models								
Inter generational	109.03	<0.0001	70.95	<0.0001	40.48	<0.0001	87.81	<0.0001
Inter generational without constraints	21.55	<0.0001	24.66	<0.0001	17.74	<0.0001	55.35	<0.0001
Intra generational	89.89	<0.0001	70.08	<0.0001	40.78	<0.0001	95.64	<0.0001
Intra generational without constraints	6.65	<0.0001	4.52	0.0013	2.23	**0.0639**	6.20	0.0001
Any mobility	102.18	<0.0001	71.89	<0.0001	38.22	<0.0001	79.28	<0.0001
Any mobility with 3-way interaction	29.44	<0.0001	20.84	<0.0001	5.45	0.0002	8.90	<0.0001

* The P-values test whether the life course model is significantly different from the saturated model. P-values in bold indicate where a model fits as well as the saturated model.

Supplementary Table 2: Results of tests comparing alternative life course hypotheses for crystallised cognitive ability (NSHD: NART score, Whitehall II: Mill Hill test score) with the saturated model, using a cutoff of very low SEP vs. the rest. NSHD models are unadjusted, Whitehall II models are adjusted for age and number of times the Mill Hill test has previously been taken. Multiple imputation is implemented to account for missing data

	NSHD				Whitehall II			
	Women (N=2,547)		Men (N=2,815)		Women (N=3,413)		Men (N=6,895)	
**Hypothesis**	F statistic	P-value*	F statistic	P-value*	F statistic	P-value*	F statistic	P-value*
No effect	72.65	<0.0001	58.47	<0.0001	103.75	<0.0001	72.88	<0.0001
Accumulation models								
Accumulation	17.97	<0.0001	15.55	<0.0001	24.03	<0.0001	18.8	<0.0001
Adult accumulation	5.97	<0.0001	0.57	**0.7258**	11.80	<0.0001	6.39	<0.0001
Sensitive period	0.38	**0.8216**	0.24	**0.9166**	6.30	0.0001	0.35	**0.8413**
Critical period models								
Childhood SEP	68.58	<0.0001	61.92	<0.0001	115.86	<0.0001	78.89	<0.0001
Early-adulthood SEP	3.36	0.0029	4.42	0.0002	73.79	<0.0001	49.68	<0.0001
Adult SEP	75.73	<0.0001	63.20	<0.0001	17.16	<0.0001	21.68	<0.0001
Social mobility models								
Inter generational	64.81	<0.0001	48.99	<0.0001	113.90	<0.0001	71.37	<0.0001
Inter generational without constraints	1.63	**0.1663**	3.66	0.0060	106.95	<0.0001	69.22	<0.0001
Intra generational	31.16	<0.0001	14.77	<0.0001	119.40	<0.0001	55.48	<0.0001
Intra generational without constraints	3.45	0.0085	2.92	0.0207	0.89	**0.4711**	2.28	**0.0607**
Any mobility	44.54	<0.0001	29.66	<0.0001	58.06	<0.0001	35.08	<0.0001
Any mobility with 3-way interaction	29.37	<0.0001	24.99	<0.0001	8.50	<0.0001	10.58	<0.0001

* The P-values test whether the life course model is significantly different from the saturated model. P-values in bold indicate where a model fits as well as the saturated model.
