# Supplementary material for: The effect of life course socioeconomic position on crystallised cognitive ability in two large UK cohort studies: a structured modelling approach
Source: BMJ Open. 2017 Jun 2;7(5):e014461. doi: 10.1136/bmjopen-2016-014461 (PMC5541359; doi:10.1136/bmjopen-2016-014461)
Supplement: Author's manuscript [file bmjopen-2016-014461.draft_revisions.pdf]

The effect of life course socioeconomic position on crystallised cognitive ability in two large UK cohort studies

Journal:	*BMJ Open*
Manuscript ID	bmjopen-2016-014461
Article Type:	Research
Date Submitted by the Author:	26-Sep-2016
Complete List of Authors:	Landy, Rebecca; Queen Mary University of London<br>Head, Jenny; UCL, Epidemiology and Public Health<br>Richards, M; UCL, MRC Unit for Lifelong Health and Ageing at UCL<br>Hardy, Rebecca; MRC Unit for Lifelong Health and Ageing
**Primary Subject Heading**:	Epidemiology
Secondary Subject Heading:	Research methods
Keywords:	socioeconomic position, cognitive function, life course, cohort studies

Note: The following files were submitted by the author for peer review, but cannot be converted to PDF. You must view these files (e.g. movies) online.
Appendix 2.doc

SCHOLARONE™ Manuscripts

For peer review only

The effect of life course socioeconomic position on crystallised cognitive ability in two large UK cohort studies

Running title: life course SEP and crystallised cognitive ability

Rebecca Landy, Jenny Head, Marcus Richards and Rebecca Hardy

Rebecca Landy (r.landy@qmul.ac.uk), Centre for Cancer Prevention, Wolfson Institute of Preventive Medicine, Bart's & The London School of Medicine, Queen Mary University of London, London, United Kingdom

Jenny Head (j.head@ucl.ac.uk), Department of Epidemiology and Public Health, University College London, London, United Kingdom

Marcus Richards (m.richards@ucl.ac.uk) and Rebecca Hardy (rebecca.hardy@ucl.ac.uk), MRC Unit for Lifelong Health and Ageing at UCL, University College London, London, United Kingdom

Correspondence to:

Rebecca Landy

Centre for Cancer Prevention

Wolfson Institute of Preventive Medicine

Charterhouse Square

London EC1M 6BQ

T: +44 20 7882 3539

F: +44 20 7882 3890

E: r.landy@qmul.ac.uk

Abstract word count: 218

Article word count: 3831

Keywords: socioeconomic position, cognitive function, life course, cohort studies

For peer review only

Strengths and limitations of the study

- Data from two large cohort studies, one population birth cohort (established 1946) and one occupational cohort (established 1985), were analysed.
- This study directly compared the relationship between life course socioeconomic position (SEP) and crystallised cognitive ability in adulthood using life course models of accumulation, sensitive period and specific forms of social mobility, rather than considering each model in isolation, which could give misleading results.
- Multiple imputation was applied to account for missing data.
- We consistently found accumulation models to be the most appropriate life course model, with comparison across a population based and occupational cohort emphasizing the importance of both relative and absolute disadvantage.
- It was necessary to select three SEP variables to represent different life course stages, and to dichotomise the SEP variables, thus losing information.

For peer review only

Abstract

This study systematically compared accumulation, critical period and social mobility models relating life course socioeconomic position (SEP) and adult crystallised cognitive ability, which has not been comprehensively investigated. The Medical Research Council (MRC) National Survey of Health and Development (NSHD) birth cohort study and the Whitehall II occupational cohort study were used to directly compare accumulation, critical period and social mobility hypotheses relating life course SEP to crystallised cognitive ability in adulthood. Childhood SEP was measured by father's occupational SEP, early-adulthood SEP by educational qualifications, and adult SEP by own occupational SEP. Each life course model was compared to a saturated model. Multiple imputation was carried out to account for missing data (NSHD: N=5,362, Whitehall II: N=10,308). The accumulation model, which contained childhood, early-adulthood and adult SEP terms, provided the best fit for both men and women in the NSHD and Whitehall II cohorts. Early-adulthood SEP had the largest coefficient in NSHD women, whereas for NSHD men early-adulthood and adult SEP had similar coefficients. In Whitehall II adult SEP had the largest effect size for both men and women. Accumulation was the most appropriate life course models for adult crystallised cognitive ability in both cohorts, including an effect of childhood SEP. It is important to directly compare the life course models to determine which is the most appropriate.

For peer review only

For peer review only - http://bmjopen.bmj.com/site/about/guidelines.xhtml

Crystallised intelligence, originally identified psychometrically by Cattell, (1) involves knowledge, such as abstract information about the world, and the meaning and pronunciation of words. It has been characterised as a mental ability that develops through the "investment" of general intelligence into learning through education and experience.(2) Crystallised ability is distinct from 'fluid' intelligence, which is the capacity to reason and solve problems in novel situations, independently of general knowledge. Whereas fluid ability is sensitive to age and morbidity-associated decline, crystallized ability accumulates over time and remains stable, or may even improve, in older age.(3)

Crystallized cognitive function is associated with health and mortality.(4) In 2009 a special issue of the journal Intelligence focussed on cognitive epidemiology, with a discussion article commenting that "social scientists and practitioners cannot afford to neglect cognitive ability when modelling epidemiological and health care phenomena". (5) Similarly, Singh-Manoux recently commented that "impaired cognitive status is one of the biggest challenges of the future due to its impact on both the individual and society".(6)

Understanding the risk factors which affect cognitive ability is therefore of importance, as such factors could represent the underlying causes of any association between cognitive ability and health. Previous findings suggest associations between higher childhood and adult socioeconomic position (SEP) and higher crystallised ability in adulthood.(7-16) It has been suggested that an individual's SEP background, including childhood SEP, may impact structural and functional brain development.(17) Childhood SEP is associated with educational qualifications gained later in life, and education in turn influences later life employment and income. However it is not clear how the trajectory of SEP throughout the life course influences crystallized cognitive function.

For peer review only - http://bmjopen.bmj.com/site/about/guidelines.xhtml

Two broad types of life course models for the influence of SEP on health outcomes have been proposed:(18) accumulation and critical period. The accumulation model proposes that exposure to socioeconomic disadvantage over the life course has a cumulative impact on the outcome. The critical period model hypothesises that SEP during a specific time window has an effect on the outcome, with no impact of SEP at any other time point. There is also interest in social mobility, but whether the social mobility model can be disentangled from the accumulation model depends on how social mobility is operationalised.(19, 20) Support for any particular life course model depends on the *a priori* hypothesis being tested. Testing only one type of model can therefore be misleading if alternative models are not also considered.(20) A structured approach to model selection proposed by Mishra et al(20) has been applied to investigate life course SEP in relation to multiple health outcomes such as osteoarthritis, allostatic load, cardiovascular risk factors, blood pressure and circulatory disease,(21-25) but has not yet been applied to crystallised ability. With increasing application of this method, challenges in model selection and interpretation of findings have become apparent (26) and an increased range of models have been parameterised.(21, 22, 25) In addition to the accumulation, critical period and social mobility models proposed by Mishra et al (20) (see details in the methods section), we consider additional accumulation type models; the 'relaxed accumulation' model, which hypothesises that SEP at each time point has an effect on the outcome(21, 23, 24), and an 'adult accumulation' model which assumes no effect of childhood SEP once early adulthood and adult SEP are included in the model. An additional social mobility model allows the outcome to differ between those who remain in the less advantaged and more advantaged SEP category for all three time points (where the original model forced them to have the same mean outcome).

For peer review only - http://bmjopen.bmj.com/site/about/guidelines.xhtml

We used the structured approach to model selection to investigate how life course SEP was associated with adult crystallised ability in two socioeconomically contrasting studies from the UK; a nationally representative general population cohort (the MRC National Survey of Health and Development (NSHD)) and an occupational cohort of civil servants (the Whitehall II study). In both studies, life course SEP has previously been related to crystallised cognitive ability, with accumulation models supported.(27, 28) The effect of inter-generational social mobility has also been supported in NSHD, with the socially mobile having cognitive levels intermediate to those in the two stable groups.(13) This finding is, however, consistent with the accumulation hypothesis where early- and late-life disadvantage have an additive effect. We directly compare multiple alternative models in order to identify the best fitting model and compare with previous findings, and evaluate the methodology used in such an approach. As less advantaged SEP and lower cognitive ability are major indicators of dropout in longitudinal studies,(29, 30) multiple imputation was carried out to account for missing data.

<u>Methods</u>

Study participants and variables

The MRC National Survey of Health and Development (NSHD), also known as the 1946 British birth cohort study, is a stratified random sample of 5,362 children born in one week in March 1946. The 22nd wave of data collection on the full cohort took place in 2006-2011,(31) though no measure of crystallised ability was collected at this wave. By age 53, the age at which crystallised ability used in this study was tested, 8.7% of participants had died, 8.6% had emigrated and 2.2% were living abroad. Compared with national data, more

For peer review only

men and women in the NSHD were employed full time at both ages 43 and 53,(32) but overall the NSHD was broadly representative.

The baseline survey of the Whitehall II study took place in 1985-1988, and recruited 10,308 non-industrial civil servants aged 35-55 (born 1930–1953).(33) Phase 11 was collected in 2012-13, with phases alternating between clinic visits and postal questionnaires. The cognitive outcome used in this study was collected at Phase 9 (2007-2009). By Phase 9 9.3% of the baseline respondents had died.

The National Adult Reading Test (NART) was administered at age 53 in NSHD, and the Mill Hill vocabulary test at Phase 9 (when participants were aged 58-74) in Whitehall II. The NART is a pronunciation test; participants are given a list of words which violate conventional grapheme-phoneme correspondence rules, and are therefore unlikely to be pronounced correctly unless the participant is familiar with the word.(34) The Mill Hill test (35) is a written vocabulary test which assesses the participants' understanding of words; participants must select the correct synonym for each word from a list of six alternatives. Both tests are well validated measures of verbal crystallised intelligence.(36, 37) To allow the results to be compared between the two studies, for each gender we standardised the NART and Mill Hill test scores.

SEP variables were dichotomised to carry out life course analyses. Childhood SEP in both studies was measured using father's occupational SEP, collected prospectively at age 4 in NSHD, and recalled retrospectively when participants were aged 44-69 in Whitehall II, categorized according to the Registrar General (RG) classification, dichotomised to manual/non-manual. Different markers of SEP in adulthood were used here, as Whitehall II is a sample of non-manual workers. Educational qualifications were the marker of early-

For peer review only

adulthood SEP, collected until age 26 for NSHD and reported retrospectively for Whitehall II. The less advantaged SEP groups were considered GCE 'O'-level (or equivalent) or below in NSHD and GCE 'A'-level/national diploma/certificate (or equivalent) or below in Whitehall II, due to the different distributions. In NSHD, adult SEP was own occupational SEP at age 43 (or age 36 if no occupation was recorded at 43), again dichotomised into manual/non-manual according to the Registrar General classification. In Whitehall II the last recorded civil service grade at phase 7 was used in the analyses, with Unified Grades 1-7 considered higher SEP. For participants no longer working in the civil service, the last recorded civil service grade was used.

Ethical approval for NSHD data collection at age 53 years was issued by North Thames Multi-centre Research Ethics Committee (MREC 98/1/121). Ethical approval for the Whitehall II study was obtained from the University College London Medical School Committee on the ethics of human research. All participants provided written informed consent.

Statistical analysis

All analyses were carried out using multiple imputation and repeated using complete cases. Since the data are not Missing Completely At Random (MCAR), multiple imputation results are presented as the primary analyses. The method of Mishra et al. compares a series of life course models with the saturated model to determine which model provides the best fit to the data.(20) The saturated model (Table 1) consists of the main effects for SEP at each of the three time points, the three two-way interactions and the single three-way interaction. This allows a different mean for each of the resulting eight possible life course SEP trajectories. Ordinary least squares multiple regression models were carried out.

For peer review only - http://bmjopen.bmj.com/site/about/guidelines.xhtml

All 10 other models considered here are nested within this saturated model, with explicit constraints on the parameters relating to different life course hypotheses. The model and constraints for each life course model tested are provided in Table 1.

For the accumulation model, the three binary SEP variables (0 for less advantaged SEP, 1 for more advantaged SEP) are summed to produce a score from 0-3. The three critical period models contain only the SEP measure at the relevant time point (childhood, early adulthood or midlife). Three types of social mobility were described by Mishra et al.:(20) inter-generational mobility, intra-generational social mobility, and any mobility.

The additional models we consider are as follows: the 'relaxed accumulation' model (21, 23, 24), in which each time point has an effect on the outcome, but the associations can differ in magnitude; an 'adult accumulation' model containing only early-adulthood and adult SEP; and an additional social mobility model containing a three-way interaction between SEP at each of the three time points, allowing the outcome to differ between those who remain in the less advantaged and more advantaged social class for all three time.

Each of the 10 life course models was tested against the saturated model using a partial F-test. If the result of a statistical test comparing two nested models is not statistically significant (p>0.05), there is no evidence the more complex model explains the data better than the simpler model; thus the simpler model is preferred. If multiple life course models were not significantly different to the saturated model, the model with the lowest Bayesian information criterion (BIC) was selected in complete case analyses. As the BIC cannot be applied with multiple imputation(38) we inspected the model coefficients to select a model.

As the Mill Hill test had been taken 0-5 times prior to phase 9 in the Whitehall II study, these analyses were adjusted for phase 9 age and the number of times the cognitive tests had

For peer review only - http://bmjopen.bmj.com/site/about/guidelines.xhtml

previously been taken. There was a significant increase in Mill Hill test score with increasing number of times the test had been taken; a linear relationship between practice effects and crystallised cognitive function provided the best fit, with the lowest BIC (not shown). Analyses were carried out separately by gender due to evidence of gender interactions with level of educational qualification (p=0.002) and occupational SEP (p=0.04) in NSHD. Multiple imputation using chained equations was carried out using the Stata command *ice*. Separate imputation models were chosen for each gender in each dataset. The imputation model was chosen following the guidelines of van Buuren (39) and Carpenter and Plewis,(40) considering a wide selection of variables from all phases of the studies. Full details of the imputation models are provided in Appendix 1. Twenty imputations were used.(41) For the multiple imputation analyses, the crystallised cognitive ability measures were standardised after imputation.

We considered an alternative cutoff for SEP, dichotomising the variables into very low SEP vs. the rest as a sensitivity analysis, with approximately the least advantaged 10-20% considered as the very low SEP category (cutoffs: NSHD: father's occupational SEP: RG categories IV and V, educational qualifications: none attempted, occupational SEP: RG category V. Whitehall II: father's occupational SEP: RG categories IV and V, educational qualifications: school matriculation or lower, occupational SEP: clerical/support jobs).

Analyses were carried out in Stata version 11.(42)

<u>Results</u>

In NSHD, adult crystallised ability at age 53 was available for 1,370 men and 1,455 women. Complete data on cognitive ability at age 53 and the three SEP variables were available for

For peer review only - http://bmjopen.bmj.com/site/about/guidelines.xhtml

1133 men (77% of those interviewed at age 53) and 1160 women (74%). Cognitive ability at Phase 9 of Whitehall II was available for 4,357 men and 1,687 women, of whom 2650 (56% of Phase 9 participants) and 900 (45%) also had complete data on the three SEP variables.

Table 2 gives the frequencies for each life course trajectory, for the complete case and multiple imputation analyses. For men the most common life course SEP trajectory was those who remained in the more advantaged category at all three time points (Table 2). For women, in NSHD the largest group was those whose father had a manual occupation, had a lower level of education, and had a non-manual occupation, whereas in Whitehall II the largest group were in the less advantaged category at all three time points. In both studies, a higher proportion of those with a missing outcome variable were in the more disadvantaged category (Table 3).

Life course analyses

In NSHD women and both genders in Whitehall II, in analyses using multiple imputation (Table 4), the relaxed accumulation model fitted the data as well as the saturated model. In NSHD men, both the relaxed accumulation model (p=0.98) and the adult accumulation model (p=0.31) fitted as well as the saturated model. Inspection of the coefficients from these models suggested an association with childhood SEP, hence the relaxed accumulation model was selected.

Although accumulation models are generally supported, the results suggest that adult SEP has a stronger influence than childhood SEP. The relaxed accumulation models indicate that adult SEP had the largest coefficient for men in both studies and women in Whitehall II. For women in NSHD, early-adulthood SEP had the largest coefficient (Table 5). For men in NSHD

For peer review only - http://bmjopen.bmj.com/site/about/guidelines.xhtml

the coefficients for early-adulthood (β=0.59 (95% CI:0.47-0.70)) and later adult SEP (β=0.65 (95% CI:0.53-0.76)) were very similar, and around 3 times the magnitude of the childhood SEP coefficient (β=0.21 (95% CI:0.10-0.32)). For women in Whitehall II, the childhood SEP coefficient was small (β=0.12 (95% CI:0.02-0.22)), with an intermediate effect of early-adulthood SEP (β=0.39 (95% CI:0.29-0.49)) compared to the effect of adult SEP (β=0.75 (95% CI:0.65-0.85)). The magnitude of the coefficients for Whitehall II men were similar to the coefficients for Whitehall II women (childhood SEP: β=0.15 (95% CI:0.09-0.21), early-adulthood SEP: β=0.37 (95% CI:0.31-0.43) and adult SEP: β=0.56 (95% CI:0.51-0.62)).

Complete case analyses

The relaxed accumulation model remained the best fit for Whitehall II women and both genders in NSHD in agreement with the main analysis. However for Whitehall II men, all models, including the relaxed accumulation model, were significantly poorer in fit than the saturated model (p=0.02) (supplementary table 1) supporting the saturated model where each trajectory has a different mean cognitive score is selected.

Sensitivity analysis

When SEP was dichotomised into very low SEP vs. the rest (supplementary table 2), the same life course models were significantly different from the saturated model except for Whitehall II women, where the relaxed accumulation model was no longer significantly different from the saturated model, implying the interaction terms were more important. The interaction between early adulthood and adult SEP remained significant (standardised β 0.40, 95% CI: 0.25-0.55), showing a larger impact on crystallised cognitive function for women who were in the more advantaged SEP category at both these time points.

For peer review only - http://bmjopen.bmj.com/site/about/guidelines.xhtml

For peer review only

Discussion

In NSHD and Whitehall II, for both genders our analyses support accumulation models suggesting more advantaged SEP at multiple points in the life course is associated with higher adult cognition. The results suggest the association with adult SEP is stronger than with childhood SEP. The consistency of these findings across the two cohorts despite differences in the measurement and distribution of adult SEP, emphasises the robustness of SEP at multiple stages of life in relation to crystallised cognitive ability, even within a limited range of SEP. The NSHD and Whitehall II study sample very different populations, with Whitehall II a selective occupational cohort with all study members in a relatively advantaged non-manual occupational group in adulthood, and less variation in SEP across the life course than the population sample; everyone in Whitehall II would be in the more advantaged adult SEP category in the NSHD. The general consistency in selection of the accumulation model across the two studies emphasizes the importance of relative disadvantage throughout the life course, as well as absolute disadvantage, across the whole SEP distribution. Differences in the relative importance of SEP at different life stages were however observed between the two cohorts. The results were more similar between NSHD and Whitehall II in men than women. Educational qualifications were more important for women in the population-based cohort than the occupational cohort which is likely to be due to the different cut-points used as the majority of women in Whitehall II stay at school past the minimum school leaving age. It is likely to have impacted the type of future jobs available to women in the population cohort, with more highly educated women in NSHD likely to enter jobs which would increase their crystallised cognitive function, explaining the stronger relationship between education and crystallised cognitive function in this cohort. Occupational SEP in adulthood was more important for women in Whitehall II than NSHD;

For peer review only - http://bmjopen.bmj.com/site/about/guidelines.xhtml

crystallized cognitive function accumulates through education and other experiences, including employment, with the type of tasks required in the civil service likely to contribute more than many other jobs held by women in a population cohort. Additionally, there is less variation in educational qualifications for women in Whitehall II, increasing the relative importance of occupational SEP as a predictor of crystallised cognitive function.

Our findings using the structured modelling approach to consider various alternative models support previous work on these cohorts, which suggests the importance of accumulation of SEP across life.(27, 28) This is also consistent with research supporting accumulation from other cohorts.(13, 43, 44) Previous support for an association with social mobility is consistent with accumulation rather than social mobility defined here in terms of effect modification, though it is not easy to disentangle accumulation and social mobility. Those experiencing upwards or downwards mobility have accumulation scores intermediate to those whose SEP is always low or always high.(13, 43, 44) This highlights the advantage of using this methodology to directly compare the life course models. These results further demonstrate that crystallised ability can be augmented across the life course, consistent with its role as a marker of accumulating knowledge and experience. Interestingly, such augmentation can be observed with other verbal domains, such as memory (13, 27) and fluency,(13) although this is less pronounced for nonverbal function.(27)

Using structural equation models, Richards and Sacker (27) and Singh-Manoux et al. (28) found that the effects of early life SEP were mediated through childhood cognition, education and adult occupation, with no direct effect remaining between childhood SEP and cognition, although direct effects were found for both education and adult occupation, suggesting an adult accumulation model. We observed a weaker association with childhood

For peer review only - http://bmjopen.bmj.com/site/about/guidelines.xhtml

than adult SEP, supporting evidence that some of the association may be mediated via later SEP. The structured approach does not test mediation models, so does not explicitly consider what proportion of the childhood SEP effect acts through later life SEP and whether any direct effect remains, though if the childhood SEP term in the relaxed accumulation model is not significant, this implies no direct effect of childhood SEP remains.

We have used a relatively new structured modelling approach and allowed for missing data using multiple imputation. The main advantage of this life course methodology is its ability to compare multiple pre-specified life course models to a saturated model, testing whether each model is sufficiently complex to describe the relationship. This is an advantage over models which consider only one life course hypothesis. However, the method is based on P-values, hence the power to detect a difference in fit between models depends on the sample size, which may explain the selection of the saturated model for Whitehall II men in the complete case analyses. Further, it is not clear which model to select when more than one model fits the data as well as the saturated model. Due to the different number of parameters in the different life course models, it is not sufficient to compare P-values. We therefore used the BIC, which penalises the model for each additional parameter fitted; hence models with lower P-values were often selected as the best fit to the data. However, with large samples the BIC tends to favour simpler models due to the heavy penalty imposed for the number of parameters in the model. In the multiple imputation analyses, where the BIC cannot be used to compare the results, the simpler model was selected if it appeared reasonable in light of the coefficients in the relevant models.(24)

Multiple imputation was carried out, as the missing data were not MCAR. Although similar conclusions were reached for complete case and multiple imputation analyses, it is important that missing data are appropriately accounted for, as estimates can be biased

For peer review only - http://bmjopen.bmj.com/site/about/guidelines.xhtml

when missing data are not MCAR yet complete case analyses are carried out. It is straightforward to apply multiple imputation to the structured modelling approach. However multiple imputation requires the assumption that data are MAR; the missing data is therefore a limitation of the study, but there is no reason to suspect that this would have a great impact on the findings.

It was necessary to select three SEP variables to represent different life course stages and to dichotomise the SEP variables, resulting in the loss of information. Selecting the life course SEP variables was more of a challenge in the Whitehall II study; the adult SEP variable was the last recorded occupational SEP at phase 7, when the participants were aged 50-74. The ages at which the 3 selected SEP variables were measured were less evenly distributed for some participants than others, and the accumulation model corresponds less well to the length of time spent in more advantaged SEP conditions. What the different measures of SEP at the different stages in the life course represent must be considered: in this study childhood SEP is measured by father's occupational SEP, which is likely to be a proxy for material circumstances as well as the environment in the home, which will in turn influence level of education.(21) Higher educational qualifications provide human capital (skills, abilities and resources), a credential for selection into the labour market (45) as well as specific skills for work, and a sense of personal control, which is associated with health in general and healthy lifestyles, as well as improving work and economic conditions.(46)

In conclusion, the accumulation hypothesis was supported when testing the relationship between life course SEP and cognitive ability in adulthood in these two contrasting cohorts, demonstrating the importance of each period of the life course, including childhood, though the relative effect sizes for each period varied by population and gender.

For peer review only - http://bmjopen.bmj.com/site/about/guidelines.xhtml

For peer review only

Acknowledgements

We thank the NSHD study members for their continuing support and members of the NSHD scientific and data collection teams, and all participating women and men in the Whitehall II Study, as well as all Whitehall II research scientists, study and data managers and clinical and administrative staff who make the study possible.

Data access

Whitehall II and NSHD data, protocols, and other metadata are available to bona fide researchers for research purposes. Please refer to the Whitehall II data sharing policy at http://www.ucl.ac.uk/whitehallII/data-sharing

and the National Survey of Health and Development data sharing policy at http://www.nshd.mrc.ac.uk/data/

Contribution statement

Rebecca Landy co-designed the study and the analytic plan, analysed the data and drafted the manuscript. Jenny Head co-designed the study's analytic plan, and critically revised the manuscript. Marcus Richards helped focus and direct the study, and critically revised the literature review and Discussion section. Rebecca Hardy co-designed the study, co-designed the study's analytic plan, and critically revised the manuscript. All authors approve the submitted version of the manuscript. All authors declare that they have no competing interests.

Funding:

RL was supported by an ESRC PhD studentship at University College London. The UK Medical Research Council, British Heart Foundation, and the US National Institutes of Health

For peer review only - http://bmjopen.bmj.com/site/about/guidelines.xhtml

(R01HL36310, R01AG013196) have supported collection of data in the Whitehall II Study. JH is partially supported by the Economic and Social Research Council (ES/K01336X/1). The MRC National Survey of Health and Development, MR and RH are funded by the UK Medical Research Council (MC_UU_12019/1, MC_UU_12019/2, MC_UU_12019/3).

For peer review only

For peer review only - http://bmjopen.bmj.com/site/about/guidelines.xhtml

Table 1: Model and constraints for each life course model tested

	Model specification	Constraints
Saturated model	$\alpha + \beta_1 S_1 + \beta_2 S_2 + \beta_3 S_3 + \theta_{12} S_1 S_2 + \theta_{23} S_2 S_3 + \theta_{13} S_1 S_3 + \theta_{123} S_1 S_2 S_3$	
No effect	α	
Accumulation models		
Accumulation	$\alpha + \beta_1 S_1 + \beta_2 S_2 + \beta_3 S_3$	$\beta_1 = \beta_2 = \beta_3$
Adult accumulation	$\alpha + \beta_2 S_2 + \beta_3 S_3$	$\beta_2 = \beta_3$
Relaxed accumulation	$\alpha + \beta_1 S_1 + \beta_2 S_2 + \beta_3 S_3$	
Critical period models		
Childhood	$\alpha + \beta_1 S_1$	
Early adulthood	$\alpha + \beta_2 S_2$	
Adulthood	$\alpha + \beta_3 S_3$	
Social mobility models		
Inter generational	$\alpha + \beta_1 S_1 + \beta_2 S_2 + \beta_{12} S_1 S_2$	$\beta_1 + \beta_2 = -\beta_{12}$
Intra generational	$\alpha + \beta_2 S_2 + \beta_3 S_3 + \beta_{23} S_2 S_3$	$\beta_2 + \beta_3 = -\beta_{23}$
Any mobility	$\alpha + \beta_1 S_1 + \beta_2 S_2 + \beta_3 S_3 + \beta_{12} S_1 S_2 + \beta_{23} S_2 S_3$	$\beta_2 = \beta_1 + \beta_3 = -\beta_{12} = -\beta_{23}$
Any mobility with 3-way interaction	$\alpha + \beta_1 S_1 + \beta_2 S_2 + \beta_3 S_3 + \beta_{12} S_1 S_2 + \beta_{23} S_2 S_3 + \beta_{123} S_1 S_2 S_3$	$\beta_2 = \beta_1 + \beta_3 = -\beta_{12} = -\beta_{23}$

S_i are the binary life course SEP variables, where S_i is equal to 0 when the participant is in the less advantaged SEP group at time point *i*, and 1 when the participant is in the more advantaged SEP group at time point *i*. The saturated model consists of the main effects for SEP at each of the three time points, as well as the three two-way interactions and the single three-way interaction.

Table 2: SEP trajectory frequencies, using father's occupational SEP (childhood SEP), educational qualifications (early-adulthood SEP) and own adult occupational SEP (adult SEP)

	NSHD		Whitehall II	
	Men	Women	Men	Women

For peer review only

Childhood SEP	Early-adulthood SEP	Adult SEP	Complete case		Multiple imputation		Complete case		Multiple imputation		Complete case		Multiple imputation		Complete case		Multiple imputation	
			N	%	N	%	N	%	N	%	N	%	N	%	N	%	N	%
0	0	0	263	23	789.8	29	226	19	565	22	454	17	1382.2	20	341	38	1510.9	44
0	0	1	175	15	476.6	17	327	28	731	29	281	11	652.4	9	25	3	67.2	2
0	1	0	55	5	98.2	4	4	0	13.8	1	87	3	235.8	3	19	2	83.4	2
0	1	1	151	13	323.6	12	90	8	172.7	7	236	9	555.2	8	24	3	58.8	2
1	0	0	62	5	181.1	4	59	5	153.7	6	499	19	1434.2	21	240	27	1005.7	29
1	0	1	111	10	295.5	11	203	18	449.5	18	367	14	849.1	12	52	6	131.8	4
1	1	0	24	2	59	2	11	1	23.1	1	165	6	489.9	7	83	9	268.1	8
1	1	1	292	26	591.3	21	240	21	438.3	17	561	21	1296.4	19	116	13	287.4	8
Total			1133	100	2815	100	1160	100	2547	100	2650	100	6895	100	900	100	3413	100

* Mean of 20 imputations. SEP=0 if the participant is in the less advantaged SEP category, and SEP=1 if the participant is in the more advantaged SEP category.

For peer review only

For peer review only - http://bmjopen.bmj.com/site/about/guidelines.xhtml

Table 3: Distribution of childhood SEP (S_1) and early- adulthood SEP (S_2) for men and women in each of the NSHD and Whitehall II study by whether the outcome variable was observed

		Men				Women			
		Observed crystallised ability		Missing crystallised ability		Observed crystallised ability		Missing crystallised ability	
Whitehall II		N	%	N	%	N	%	N	%
S_1	0	1189	40	792	45	516	47	619	56
	1	1814	60	979	55	591	53	480	44
S_2	0	2273	59	637	65	974	71	443	81
	1	1569	41	344	35	398	29	102	19
NSHD									
S_1	0	720	57	680	62	749	56	517	64
	1	543	43	412	38	583	44	296	36
S_2	0	719	55	695	69	974	71	593	80
	1	582	45	312	31	405	29	152	20

For peer review only - http://bmjopen.bmj.com/site/about/guidelines.xhtml

For peer review only

Table 4: Results of tests comparing alternative life course hypotheses for crystallised cognitive ability (NSHD: NART score, Whitehall II: Mill Hill test score) with the saturated model (NSHD models are unadjusted, Whitehall II models are adjusted for age and number of times the Mill Hill test has previously been taken. Multiple imputation is implemented to account for missing data)

	NSHD				Whitehall II			
	Women (N=2,547)		Men (N=2,815)		Women (N=3,413)		Men (N=6,895)	
**Hypothesis**	F statistic	P-value*	F statistic	P-value*	F statistic	P-value*	F statistic	P-value*
No effect	94.00	<0.0001	86.99	<0.0001	58.98	<0.0001	129.45	<0.0001
Accumulation models								
Accumulation	6.11	<0.0001	7.05	<0.0001	13.44	<0.0001	18.03	<0.0001
Adult accumulation	5.64	<0.0001	1.19	**0.3116**	4.28	0.0003	7.89	<0.0001
Relaxed accumulation	1.39	**0.2375**	0.10	**0.9821**	0.97	**0.4224**	0.32	**0.8670**
Critical period models								
Childhood SEP	63.58	<0.0001	77.04	<0.0001	61.78	<0.0001	138.57	<0.0001
Early-adulthood SEP	19.95	<0.0001	32.40	<0.0001	30.50	<0.0001	72.73	<0.0001
Adult SEP	61.93	<0.0001	32.94	<0.0001	12.21	<0.0001	34.71	<0.0001
Social mobility models								
Inter generational	117.88	<0.0001	111.71	<0.0001	82.01	<0.0001	171.74	<0.0001
Intra generational	117.00	<0.0001	123.12	<0.0001	64.91	<0.0001	174.27	<0.0001
Any mobility	128.51	<0.0001	112.79	<0.0001	69.45	<0.0001	152.22	<0.0001
Any mobility with 3-way interaction	31.25	<0.0001	25.20	<0.0001	9.28	<0.0001	28.91	<0.0001

* The P-values test whether the life course model is significantly different from the saturated model. P-values in bold indicate where a model fits as well as the saturated model

For peer review only - http://bmjopen.bmj.com/site/about/guidelines.xhtml

Table 5: Standardised coefficients for each term in the relaxed accumulation models, in the NSHD and Whitehall II, by gender.

	Men				Women			
	Complete Case		Multiple imputation		Complete Case		Multiple imputation	
	Coeff	95% CI	Coeff	95% CI	Coeff	95% CI	Coeff	95% CI
	Unadjusted NSHD				Unadjusted NSHD			
Childhood SEP	0.23	(0.11, 0.35)	0.21	(0.10, 0.32)	0.32	(0.19, 0.44)	0.36	(0.25, 0.48)
Early-adulthood SEP	0.51	(0.38, 0.64)	0.59	(0.47, 0.70)	0.83	(0.70, 0.96)	0.82	(0.71, 0.93)
Adult SEP	0.66	(0.52, 0.80)	0.65	(0.53, 0.76)	0.51	(0.36, 0.65)	0.50	(0.36, 0.63)
Constant	-0.74	(-0.85, -0.64)	-0.70	(-0.79, -0.60)	-0.79	(-0.91, -0.67)	-0.72	(-0.82, -0.62)
	Whitehall II*				Whitehall II			
Childhood SEP			0.15	(0.09, 0.21)	0.14	(0.02, 0.26)	0.12	(0.02, 0.22)
Early-adulthood SEP			0.37	(0.31, 0.43)	0.43	(0.28, 0.57)	0.39	(0.29, 0.49)
Adult SEP			0.56	(0.51, 0.62)	0.62	(0.47, 0.77)	0.75	(0.65, 0.85)
Age			0.00	(-0.01, 0.00)	-0.01	(-0.02, -0.00)	-0.02	(-0.02, -0.01)
Number of times previously taken cognitive tests			0.07	(0.03, 0.10)	0.06	(-0.01, 0.14)	0.13	(0.09, 0.18)
Constant			-0.44	(-0.73, -0.14)	0.38	(-0.30, 1.05)	0.68	(0.22, 1.14)

* None of the life course models considered fit the data as well as the saturated model in the complete case analysis of men in Whitehall II.

For peer review only

Supplementary Table 1: Complete case results of tests comparing alternative life course hypotheses for crystallised cognitive ability (NSHD: NART score, Whitehall II: Mill Hill test score) with the saturated model (NSHD models are unadjusted, Whitehall II models are adjusted for age and number of times the Mill Hill test has previously been taken.

	NSHD				Whitehall II			
	Women		Men		Women		Men	
**Hypothesis**	F	P-	F	P-	F	P-	F	P-
No effect	80.72	<0.0001	53.99	<0.0001	31.16	<0.0001	70.33	<0.0001
Accumulation models								
Accumulation	4.90	0.0001	4.37	0.0002	4.74	0.0001	12.32	<0.0001
Adult accumulation	7.18	<0.0001	3.24	0.0037	1.85	**0.0861**	5.73	<0.0001
Relaxed accumulation	2.02	**0.0891**	0.69	**0.5967**	0.93	**0.4459**	2.80	0.0248
Critical period models								
Childhood	51.80	<0.0001	45.41	<0.0001	28.49	<0.0001	74.81	<0.0001
Early adulthood	23.43	<0.0001	21.63	<0.0001	13.89	<0.0001	40.90	<0.0001
Adulthood	52.01	<0.0001	21.11	<0.0001	8.55	<0.0001	19.16	<0.0001
Social mobility models								
Inter generational	109.03	<0.0001	70.95	<0.0001	40.48	<0.0001	87.81	<0.0001
Intra generational	89.89	<0.0001	70.08	<0.0001	40.78	<0.0001	95.64	<0.0001
Any mobility	102.18	<0.0001	71.89	<0.0001	38.22	<0.0001	79.28	<0.0001
Any mobility with 3-way interaction	29.44	<0.0001	20.84	<0.0001	5.45	0.0002	8.90	<0.0001

* The P-values test whether the life course model is significantly different from the saturated model. P-values in bold indicate where a model fits as well as the saturated model.

For peer review only

Supplementary Table 2: Results of tests comparing alternative life course hypotheses for crystallised cognitive ability (NSHD: NART score, Whitehall II: Mill Hill test score) with the saturated model, using a cutoff of very low SEP vs. the rest. NSHD models are unadjusted, Whitehall II models are adjusted for age and number of times the Mill Hill test has previously been taken. Multiple imputation is implemented to account for missing data

Hypothesis	NSHD				Whitehall II			
	Women (N=2,547)		Men (N=2,815)		Women (N=3,413)		Men (N=6,895)	
	F statistic	P-value*	F statistic	P-value*	F statistic	P-value*	F statistic	P-value*
No effect	72.65	<0.0001	58.47	<0.0001	103.75	<0.0001	72.88	<0.0001
Accumulation models								
Accumulation	17.97	<0.0001	15.55	<0.0001	24.03	<0.0001	18.8	<0.0001
Adult accumulation	5.97	<0.0001	0.57	**0.7258**	11.80	<0.0001	6.39	<0.0001
Relaxed accumulation	0.38	**0.8216**	0.24	**0.9166**	6.30	0.0001	0.35	**0.8413**
Critical period models								
Childhood SEP	68.58	<0.0001	61.92	<0.0001	115.86	<0.0001	78.89	<0.0001
Early-adulthood SEP	3.36	0.0029	4.42	0.0002	73.79	<0.0001	49.68	<0.0001
Adult SEP	75.73	<0.0001	63.20	<0.0001	17.16	<0.0001	21.68	<0.0001
Social mobility models								
Inter generational	64.81	<0.0001	48.99	<0.0001	113.90	<0.0001	71.37	<0.0001
Intra generational	31.16	<0.0001	14.77	<0.0001	119.40	<0.0001	55.48	<0.0001
Any mobility	44.54	<0.0001	29.66	<0.0001	58.06	<0.0001	35.08	<0.0001
Any mobility with 3-way interaction	29.37	<0.0001	24.99	<0.0001	8.50	<0.0001	10.58	<0.0001

For peer review only

For peer review only - http://bmjopen.bmj.com/site/about/guidelines.xhtml

1. Cattell RB. Abilities: Their structure, growth, and action. New York: Houghton Mifflin; 1971.
2. Carroll JB. Human cognitive abilities: a survey of factor analytic studies. Cambridge UK: Cambridge University Press; 1993.
3. Deary IJ, Batty GD. Cognitive epidemiology. J Epidemiol Community Health. 2007;61(5):378-84.
4. B. Bosworth KWS, Hayden. Survival effects in cognitive function, cognitive style, and sociodemographic variables in the Seattle Longitudinal Study. Experimental Aging Research. 1999;25(2):121-39.
5. Lubinski D. Cognitive epidemiology: With emphasis on untangling cognitive ability and socioeconomic status. Intelligence. 2009;37(6):625-33.
6. Singh-Manoux A. Commentary: Is it time to redefine cognitive epidemiology? International journal of epidemiology. 2010:dyq123.
7. Lee S, Kawachi I, Berkman LF, Grodstein F. Education, other socioeconomic indicators, and cognitive function. Am J Epidemiol. 2003;157(8):712-20.
8. Wilson R, Scherr PA, Bienias JL, Mendes de Leon CF, Everson-Rose SA, Bennett D, et al. Socioeconomic characteristics of the community in childhood and cognition in old age. Experimental aging research. 2005;31(4):393-407.
9. Zhang Z, Plassman B, Xu Q, Zahner G, Wu B, Gai M, et al. Lifespan influences on mid-to late-life cognitive function in a Chinese birth cohort. Neurology. 2009;73(3):186-94.
10. Cagney KA, Lauderdale DS. Education, wealth, and cognitive function in later life. The Journals of Gerontology Series B: Psychological Sciences and Social Sciences. 2002;57(2):P163-P72.
11. Rabbitt P, Chetwynd A, McInnes L. Do clever brains age more slowly? Further exploration of a nun result. British Journal of Psychology. 2003;94(1):63-71.
12. Gallacher JE, Elwood PC, Hopkinson C, Rabbitt PM, Stollery BT, Sweetnam PM, et al. Cognitive function in the Caerphilly study: associations with age, social class, education and mood. European journal of epidemiology. 1999;15(2):161-9.
13. Hatch SL, Feinstein L, Link BG, Wadsworth MEJ, RichardS M. The continuing benefits of education: Adult education and midlife cognitive ability in the British 1946 birth cohort. Journals of Gerontology Series B-Psychological Sciences and Social Sciences. 2007;62(6):S404-S14.
14. Kaplan GA, Turrell G, Lynch JW, Everson SA, Helkala E-L, Salonen JT. Childhood socioeconomic position and cognitive function in adulthood. International Journal of Epidemiology. 2001;30(2):256-63.
15. Everson-Rose SA, de Leon CFM, Bienias JL, Wilson RS, Evans DA. Early life conditions and cognitive functioning in later life. American Journal of Epidemiology. 2003;158(11):1083-9.
16. Turrell G, Lynch JW, Kaplan GA, Everson SA, Helkala EL, Kauhanen J, et al. Socioeconomic position across the lifecourse and cognitive function in late middle age. J Gerontol B Psychol Sci Soc Sci. 2002;57(1):S43-51.
17. Tomalski P, Johnson MH. The effects of early adversity on the adult and developing brain. Current opinion in psychiatry. 2010;23(3):233-8.
18. Ben-Shlomo Y, Kuh D. A life course approach to chronic disease epidemiology: conceptual models, empirical challenges and interdisciplinary perspectives. International journal of epidemiology. 2002;31(2):285-93.
19. Hallqvist J, Lynch J, Bartley M, Lang T, Blane D. Can we disentangle life course processes of accumulation, critical period and social mobility? An analysis of disadvantaged socio-economic positions and myocardial infarction in the Stockholm Heart Epidemiology Program. Social science & medicine. 2004;58(8):1555-62.
20. Mishra G, Nitsch D, Black S, De Stavola B, Kuh D, Hardy R. A structured approach to modelling the effects of binary exposure variables over the life course. International journal of epidemiology. 2009;38(2):528-37.

For peer review only - http://bmjopen.bmj.com/site/about/guidelines.xhtml

21. Robertson T, Popham F, Benzeval M. Socioeconomic position across the lifecourse & allostatic load: data from the West of Scotland Twenty-07 cohort study. BMC public health. 2014;14(1):184.
22. Howe LD, Chaturvedi N, Lawlor DA, Ferreira DL, Fraser A, Smith GD, et al. Rapid increases in infant adiposity and overweight/obesity in childhood are associated with higher central and brachial blood pressure in early adulthood. Journal of hypertension. 2014;32(9):1789.
23. Mishra GD, Chiesa F, Goodman A, De Stavola B, Koupil I. Socio-economic position over the life course and all-cause, and circulatory diseases mortality at age 50-87 years: results from a Swedish birth cohort. European journal of epidemiology. 2013;28(2):139-47.
24. Murray ET, Mishra GD, Kuh D, Guralnik J, Black S, Hardy R. Life course models of socioeconomic position and cardiovascular risk factors: 1946 birth cohort. Annals of Epidemiology. 2011;21(8):589-97.
25. Jones R, Hardy R, Sattar N, Deanfield JE, Hughes A, Kuh D, et al. Novel coronary heart disease risk factors at 60-64 years and life course socioeconomic position: The 1946 British birth cohort. Atherosclerosis. 2015;238(1):70.
26. Ben-Shlomo Y, Mishra G, Kuh D. Life course epidemiology. In: Ahrens W, Pigeot I, editors. Handbook of Epidemiology second ed: Springer; 2013.
27. Richards M, Sacker A. Lifetime antecedents of cognitive reserve. Journal of Clinical and Experimental Neuropsychology. 2003;25(5):614-24.
28. Singh-Manoux A, Richards M, Marmot M. Socioeconomic position across the lifecourse: How does it relate to cognitive function in mid-life? Annals of Epidemiology. 2005;15(8):572-8.
29. Richards M, Shipley B, Fuhrer R, Wadsworth ME. Cognitive ability in childhood and cognitive decline in mid-life: longitudinal birth cohort study. Bmj. 2004;328(7439):552.
30. Matthews FE, Chatfield M, Freeman C, McCracken C, Brayne C, CFAS M. Attrition and bias in the MRC cognitive function and ageing study: an epidemiological investigation. BMC public health. 2004;4(1):12.
31. Kuh D, Pierce M, Adams J, Deanfield J, Ekelund U, Friberg P, et al. Cohort profile: updating the cohort profile for the MRC National Survey of Health and Development: a new clinic-based data collection for ageing research. International journal of epidemiology. 2011;40(1):e1-e9.
32. Wadsworth M, Butterworth S, Hardy R, Kuh D, Richards M, Langenberg C, et al. The life course prospective design: an example of benefits and problems associated with study longevity. Social science & medicine. 2003;57(11):2193-205.
33. Ferrie JE, Kivimaki M, Singh-Manoux A, Shortt A, Martikainen P, Head J, et al. Non-response to baseline, non-response to follow-up and mortality in the Whitehall II cohort. International journal of epidemiology. 2009;38(3):831-7.
34. Nelson HE, Willison JR. National Adult Reading Test (NART) (2nd ed.): Windsor: NFER Nelson; 1991.
35. Raven J. Guide to Using the Mill Hill Vocabulary Scale with the Progressive Matrices Scales. Oxford, England: H.K. Lewis & Co.; 1958.
36. Crawford JR, Deary IJ, Starr J, Whalley LJ. The NART as an index of prior intellectual functioning: a retrospective validity study covering a 66-year interval. Psychological medicine. 2001;31(03):451-8.
37. Franzen M.D. Reliability and Validity in Neuropsychological Assessment. second ed: Springer Science & Business Media; 2013.
38. Statacorp. Stata help file for mim command. 2011.
39. Van Buuren S, Boshuizen HC, Knook DL. Multiple imputation of missing blood pressure covariates in survival analysis. Statistics in medicine. 1999;18(6):681-94.
40. Carpenter J, Plewis I. Analysing Longitudinal Studies with Non-response: Issues and statistical methods. In: Willians M, Vogt W, editors. The SAGE Handbook of Innovation in Social Research Methods: SAGE Publications Ltd; 2011.

For peer review only

For peer review only - http://bmjopen.bmj.com/site/about/guidelines.xhtml

41. Sterne JA, White IR, Carlin JB, Spratt M, Royston P, Kenward MG, et al. Multiple imputation for missing data in epidemiological and clinical research: potential and pitfalls. Bmj. 2009;338.
42. Statacorp. Stata Statistical Software: Release 11. College Station, TX: Statacorp LP; 2009.
43. Turrell G, Lynch JW, Kaplan GA, Everson SA, Helkala E-L, Kauhanen J, et al. Socioeconomic position across the lifecourse and cognitive function in late middle age. The Journals of Gerontology Series B: Psychological Sciences and Social Sciences. 2002;57(1):S43-S51.
44. Luo Y, Waite LJ. The impact of childhood and adult SES on physical, mental, and cognitive well-being in later life. The Journals of Gerontology Series B: Psychological Sciences and Social Sciences. 2005;60(2):S93-S101.
45. Collins R. The credential society: An historical sociology of education and stratification. New York, NY: Academic Press; 1979.
46. Ross CE, Mirowsky J. Refining the association between education and health: The effects of quantity, credential, and selectivity. Demography. 1999;36(4):445-60.

For peer review only

Appendix 1

Multiple imputation was carried out using the Stata command *ice*, created by Patrick Royston (118;119), which samples from the posterior predictive distribution $p(Y_{missing} | Y_{observed}, X)$. For 20 imputations, 20 independent selections are made from the posterior predictive distribution. All the variables which are thought to predict or be associated with the missing values should be included in the model used to form the posterior predictive distribution, the imputation model. When choosing which variables to include in the imputation model, it is important to also include any variables that may be important in subsequent analyses, including the variable(s) with missing data and the outcome of interest. It has been shown that including as many explanatory variables in the imputation model as possible makes the MAR assumption more plausible (van Buuren et al., 1999). Although including redundant predictors may be expected to reduce the precision of the final estimates, Kenward and Carpenter (Kenward and Carpenter, 2007) note that this effect is typically not large, concluding that it is better to err on the side of including too many variables in the imputation model rather than too few, as excluding an important predictor of missingness could cause bias.

van Buuren (van Buuren et al., 1999) and Carpenter and Plewis (Carpenter and Plewis, 2011) suggest similar methods for selecting the variables to include in the imputation model from a large database:

1. Include all variables that appear in the complete-data model of interest.
2. Include the variables which were predictive of missing data.
3. Check whether the variables included in step 2 were associated with the variables in the model of interest.
4. Auxiliary variables can also be included to make the assumption of MAR more likely. Auxiliary variables are predictive of any of the variables in the imputation model.

Binary variables were imputed using logistic regression, ordered categorical variables using ordered logistic regression, and unordered categorical variables using multinomial regression.

For peer review only

Variables included in the imputation model for Whitehall II men, in addition to those in the model of interest

Phase of data collection	Variables
1	Year of birth, longstanding illness, current cigarette smoker, age when finished full time education, isolation scale score, usually pressed for time, believe no one cares much about you, state of health in the last year, accommodation type, mother's age when finished education
3	Job involves travel away from home, marital status, last recorded civil service grade, memory score, AH4 score, verbal fluency (S-words, animals), Mill Hill score
4	Ever told had anxiety, ever told had depression,
5	Childhood emotional deprivation, childhood material deprivation, ever told high blood pressure, deprivation score, feel might as well give up, financial security in the next 10 years, last recorded civil service grade, memory score, AH4 score, verbal fluency (S-words, animals), Mill Hill score
7	Clinic or home visit, still working as a civil servant, general health, CASP total score, my health stops me doing things I want to do, MMSE total score, memory score, AH4 score, verbal fluency (S-words, animals), Mill Hill score
9	General health, difficulty paying bills, MMSE total score, marital status, last recorded civil service grade, memory score, AH4 score, verbal fluency (S-words, animals)

For peer review only

Variables included in the imputation model for Whitehall II women, in addition to those in the model of interest

Phase of data collection	Variables
1	Year of birth, longstanding illness, current cigarette smoker, age when finished full time education, isolation scale score, usually pressed for time, state of health in the last year, accommodation type, mother's age when finished education, satisfied by job as a whole, civil service grade
3	Job involves travel away from home, marital status, last recorded civil service grade, memory score, AH4 score, verbal fluency (S-words, animals), Mill Hill score
4	Ever told had depression
5	Childhood emotional deprivation, childhood material deprivation, ever told high blood pressure, feel might as well give up, last recorded civil service grade, memory score, AH4 score, verbal fluency (S-words, animals), Mill Hill score
7	Clinic or home visit, still working as a civil servant, general health, my health stops me doing things I want to do, MMSE total score, memory score, AH4 score, verbal fluency (S-words, animals), Mill Hill score
9	General health, difficulty paying bills, MMSE total score, marital status, last recorded civil service grade, memory score, AH4 score, verbal fluency (S-words, animals)

For peer review only

Variables included in the imputation model for NSHD men, in addition to those in the model of interest

Participant's Age	Variables
0	Father's occupational SEP
2	Childhood household amenities
4	Child has own bed, clothes repair, yard or garden, child's shoes, cleanliness of house, cleanliness of child, mother's management and understanding of the child, crowding, repair of dwelling
8	Reading score, sentence completion score, picture intelligence score, vocabulary score
10	Difficult to discipline
11	Father's occupational SEP, non-verbal reasoning score, reading score, verbal reasoning score, arithmetic score, vocabulary score, household amenities
13	Have to stick up for myself
15	Non-verbal reasoning score, reading score, verbal reasoning score, mathematics score, truancy during the past year
26	Own occupational SEP, happiness, job stress, brought up in a faith, currently have a religion
36	Marital status, current smoking status, ever been unemployed, own occupational SEP, ever had cancer, does religious upbringing effect life now
43	How many friends or relatives could you visit at any time without waiting for an invite, verbal memory score
53	Verbal fluency score

For peer review only

Variables included in the imputation model for NSHD women, in addition to those in the model of interest

Participant's Age	Variables
0	Father's occupational SEP
2	Childhood household amenities
4	Child has own bed, clothes repair, yard or garden, child's shoes, cleanliness of house, cleanliness of child, mother's management and understanding of the child, crowding, repair of dwelling, age of dwelling
8	Reading score, sentence completion score, picture intelligence score, vocabulary score
10	Difficult to discipline
11	Father's occupational SEP, non-verbal reasoning score, reading score, verbal reasoning score, arithmetic score, vocabulary score, household amenities
13	Have to stick up for myself
15	Non-verbal reasoning score, reading score, verbal reasoning score, mathematics score, truancy during the past year
26	Own occupational SEP, happiness, job stress, brought up in a faith, currently have a religion
31	Hospital admissions
36	Marital status, current smoking status, ever been unemployed, own occupational SEP, ever had cancer, does religious upbringing effect life now, blood pressure, how confident feel in myself
43	Head of household SEP, verbal memory score
53	Verbal fluency score

References:

CARPENTER, J. & PLEWIS, I. (2011) Analysing Longitudinal Studies with Non-response: Issues and statistical methods. IN WILLIANS, M. & VOGT, W. (Eds.) *The SAGE Handbook of Innovation in Social Research Methods.* SAGE Publications Ltd.

KENWARD, M. G. & CARPENTER, J. (2007) Multiple imputation: current perspectives. *Statistical Methods in Medical Research,* 16**,** 199-218.

VAN BUUREN, S., BOSHUIZEN, H. C. & KNOOK, D. L. (1999) Multiple imputation of missing blood pressure covariates in survival analysis. *Statistics in medicine,* 18**,** 681-694.

For peer review only

STROBE Statement—Checklist of items that should be included in reports of ***cohort studies***

	Item No	Recommendation	Page
**Title and abstract**	1	(*a*) Indicate the study's design with a commonly used term in the title or the abstract	1
		(*b*) Provide in the abstract an informative and balanced summary of what was done and what was found	3
**Introduction**			
Background/rationale	2	Explain the scientific background and rationale for the investigation being reported	4-5
Objectives	3	State specific objectives, including any prespecified hypotheses	5
**Methods**			
Study design	4	Present key elements of study design early in the paper	5-6
Setting	5	Describe the setting, locations, and relevant dates, including periods of recruitment, exposure, follow-up, and data collection	5-6
Participants	6	(*a*) Give the eligibility criteria, and the sources and methods of selection of participants. Describe methods of follow-up	5-6
		(*b*) For matched studies, give matching criteria and number of exposed and unexposed	n/a
Variables	7	Clearly define all outcomes, exposures, predictors, potential confounders, and effect modifiers. Give diagnostic criteria, if applicable	6-7,9
Data sources/ measurement	8*	For each variable of interest, give sources of data and details of methods of assessment (measurement). Describe comparability of assessment methods if there is more than one group	6-7
Bias	9	Describe any efforts to address potential sources of bias	7
Study size	10	Explain how the study size was arrived at	5-6
Quantitative variables	11	Explain how quantitative variables were handled in the analyses. If applicable, describe which groupings were chosen and why	6-7
Statistical methods	12	(*a*) Describe all statistical methods, including those used to control for confounding	7-9
		(*b*) Describe any methods used to examine subgroups and interactions	n/a
		(*c*) Explain how missing data were addressed	7
		(*d*) If applicable, explain how loss to follow-up was addressed	7
		(*e*) Describe any sensitivity analyses	7
**Results**			
Participants	13*	(a) Report numbers of individuals at each stage of study—eg numbers potentially eligible, examined for eligibility, confirmed eligible, included in the study, completing follow-up, and analysed	5-6
		(b) Give reasons for non-participation at each stage	5-6
		(c) Consider use of a flow diagram	n/a
Descriptive data	14*	(a) Give characteristics of study participants (eg demographic, clinical, social) and information on exposures and potential confounders	16-17
		(b) Indicate number of participants with missing data for each variable of interest	16
		(c) Summarise follow-up time (eg, average and total amount)	5-6
Outcome data	15*	Report numbers of outcome events or summary measures over time	6,17
Main results	16	(*a*) Give unadjusted estimates and, if applicable, confounder-adjusted estimates and their precision (eg, 95% confidence interval). Make clear	19

For peer review only - http://bmjopen.bmj.com/site/about/guidelines.xhtml

		which confounders were adjusted for and why they were included	
		(*b*) Report category boundaries when continuous variables were categorized	6-7
		(*c*) If relevant, consider translating estimates of relative risk into absolute risk for a meaningful time period	n/a
Other analyses	17	Report other analyses done—eg analyses of subgroups and interactions, and sensitivity analyses	n/a
**Discussion**			
Key results	18	Summarise key results with reference to study objectives	11
Limitations	19	Discuss limitations of the study, taking into account sources of potential bias or imprecision. Discuss both direction and magnitude of any potential bias	13
Interpretation	20	Give a cautious overall interpretation of results considering objectives, limitations, multiplicity of analyses, results from similar studies, and other relevant evidence	14
Generalisability	21	Discuss the generalisability (external validity) of the study results	11
**Other information**			
Funding	22	Give the source of funding and the role of the funders for the present study and, if applicable, for the original study on which the present article is based	15

*Give information separately for exposed and unexposed groups.

Note: An Explanation and Elaboration article discusses each checklist item and gives methodological background and published examples of transparent reporting. The STROBE checklist is best used in conjunction with this article (freely available on the Web sites of PLoS Medicine at http://www.plosmedicine.org/, Annals of Internal Medicine at http://www.annals.org/, and Epidemiology at http://www.epidem.com/). Information on the STROBE Initiative is available at http://www.strobe-statement.org.

For peer review only

BMJ Open

The effect of life course socioeconomic position on crystallised cognitive ability in two large UK cohort studies: a structured modelling approach

Journal:	*BMJ Open*
Manuscript ID	bmjopen-2016-014461.R1
Article Type:	Research
Date Submitted by the Author:	14-Mar-2017
Complete List of Authors:	Landy, Rebecca; Queen Mary University of London<br>Head, Jenny; UCL, Epidemiology and Public Health<br>Richards, M; UCL, MRC Unit for Lifelong Health and Ageing at UCL<br>Hardy, Rebecca; MRC Unit for Lifelong Health and Ageing
**Primary Subject Heading**:	Epidemiology
Secondary Subject Heading:	Research methods
Keywords:	socioeconomic position, cognitive function, life course, cohort studies

SCHOLARONE™ Manuscripts

For peer review only

The effect of life course socioeconomic position on crystallised cognitive ability in two large UK cohort studies: a structured modelling approach

Running title: life course SEP and crystallised cognitive ability

Rebecca Landy, Jenny Head, Marcus Richards and Rebecca Hardy

Rebecca Landy (r.landy@qmul.ac.uk), Centre for Cancer Prevention, Wolfson Institute of Preventive Medicine, Bart's & The London School of Medicine, Queen Mary University of London, London, United Kingdom

Jenny Head (j.head@ucl.ac.uk), Department of Epidemiology and Public Health, University College London, London, United Kingdom

Marcus Richards (m.richards@ucl.ac.uk) and Rebecca Hardy (rebecca.hardy@ucl.ac.uk), MRC Unit for Lifelong Health and Ageing at UCL, University College London, London, United Kingdom

Correspondence to:

Rebecca Landy

Centre for Cancer Prevention

Wolfson Institute of Preventive Medicine

Charterhouse Square

London EC1M 6BQ

T: +44 20 7882 3539

F: +44 20 7882 3890

E: r.landy@qmul.ac.uk

Abstract word count: 218

Article word count: 4562

Keywords: socioeconomic position, cognitive function, life course, cohort studies

For peer review only

Abstract

Objectives: This study systematically compared accumulation, sensitive period, critical period and social mobility models relating life course socioeconomic position (SEP) and adult crystallised cognitive ability, which has not been comprehensively investigated.

Design: Two prospective cohort studies.

Participants: 5362 participants in the Medical Research Council (MRC) National Survey of Health and Development (NSHD) birth cohort study and 10,308 participants in the Whitehall II occupational cohort study.

Measures: Childhood SEP was measured by father's occupational SEP, early-adulthood SEP by educational qualifications, and adult SEP by own occupational SEP. Each life course model was compared to a saturated model.

Results: Using multiple imputation to account for missing data, the sensitive period model, which contained childhood, early-adulthood and adult SEP terms, with different coefficients, provided the best fit for both men and women in the NSHD and Whitehall II cohorts. Early-adulthood SEP had the largest coefficient in NSHD women, whereas for NSHD men early-adulthood and adult SEP had similar coefficients. In Whitehall II adult SEP had the largest effect size for both men and women.

Conclusions: Sensitive period with all three time periods was the most appropriate life course models for adult crystallised cognitive ability in both cohorts, including an effect of childhood SEP. It is important to directly compare the life course models to determine which is the most appropriate.

For peer review only

For peer review only - http://bmjopen.bmj.com/site/about/guidelines.xhtml

Strengths and limitations of the study

- Data from two large cohort studies, one population birth cohort (established 1946) and one occupational cohort (established 1985), were analysed.
- This study directly compared the relationship between life course socioeconomic position (SEP) and crystallised cognitive ability in adulthood using life course models of accumulation, sensitive period, critical period and specific forms of social mobility, rather than considering each model in isolation, which could give misleading results.
- Multiple imputation was applied to account for missing data.
- It was necessary to select three SEP variables to represent different life course stages, and to dichotomise the SEP variables, thus losing information.

For peer review only

For peer review only - http://bmjopen.bmj.com/site/about/guidelines.xhtml

Introduction

Crystallised intelligence, originally identified psychometrically by Cattell, (1) involves knowledge, such as abstract information about the world, and the meaning and pronunciation of words. It has been characterised as a mental ability that develops through the "investment" of general intelligence into learning through education and experience.(2) Crystallised ability is distinct from 'fluid' intelligence, which is the capacity to reason and solve problems in novel situations, independently of general knowledge. Whereas fluid ability is sensitive to age and morbidity-associated decline, crystallized ability accumulates over time and remains stable, or may even improve, in older age.(3)

Crystallized cognitive function is associated with health and mortality.(4) In 2009 a special issue of the journal Intelligence focussed on cognitive epidemiology, with a discussion article commenting that "social scientists and practitioners cannot afford to neglect cognitive ability when modelling epidemiological and health care phenomena". (5) Similarly, Singh-Manoux recently commented that "impaired cognitive status is one of the biggest challenges of the future due to its impact on both the individual and society".(6)

Understanding the risk factors which affect cognitive ability is therefore of importance, as such factors could represent the underlying causes of any association between cognitive ability and health. Previous studies investigating the relationship between life course SEP and crystallized cognitive function in adulthood have tended to focus on one life course hypothesis, or if multiple hypotheses were considered, the hypotheses were not compared. For example, a dose-response relationship has been found between the amount of time spent in a more advantaged SEP and crystallised cognitive function.(7, 8) Similarly, participants who were socially mobile were found to have cognitive scores between those who remained in a low SEP and those who remained in a

For peer review only - http://bmjopen.bmj.com/site/about/guidelines.xhtml

high SEP.(7-9) Numerous studies have shown that low SEP at one point in time, whether childhood (10-14), or adulthood (9, 15-17) is associated with lower crystallised cognitive function in older age. It has been suggested that an individual's SEP background, including childhood SEP, may impact structural and functional brain development.(18) Childhood SEP is associated with educational qualifications gained later in life, and education in turn influences later life employment and income. However it is not clear how the trajectory of SEP throughout the life course influences crystallized cognitive function.

Two broad types of life course models for the influence of SEP on health outcomes have been proposed:(19) accumulation and critical period. The accumulation model proposes that exposure to socioeconomic disadvantage over the life course has a cumulative impact on the outcome. The critical period model hypothesises that SEP during a specific time window has an effect on the outcome, with no impact of SEP at any other time point. The sensitive period model suggests that SEP has a stronger association within a specific window but still has an effect outside the window. Initially, the critical or sensitive period models were described as distinct from the accumulation model, but more recently the critical and sensitive period models have been seen as special subtypes of the accumulation model. (20) There is also interest in social mobility, but whether the social mobility model can be disentangled from the accumulation model depends on how social mobility is operationalised.(21, 22) Support for any particular life course model depends on the *a priori* hypothesis being tested. Testing only one type of model can therefore be misleading if alternative models are not also considered,(22) as the data may support more than one of the life course hypotheses; it is therefore important to compare all the life course models to ensure the one which provides the best support is chosen. These exploratory analyses can then be used as a first step towards carrying out more robust analyses exploring causal

For peer review only - http://bmjopen.bmj.com/site/about/guidelines.xhtml

relationships, by identifying the most appropriate life course models to investigate further.(23, 24)

A structured approach to model selection proposed by Mishra et al(22) has been applied to investigate life course SEP in relation to multiple health outcomes such as osteoarthritis, allostatic load, cardiovascular risk factors, blood pressure and circulatory disease,(23, 25-28) but has not yet been applied to crystallised cognitive ability. With increasing application of this method, challenges in model selection and interpretation of findings have become apparent (29) and an increased range of models have been parameterised.(23, 25, 28) In addition to the accumulation, critical period and social mobility models proposed by Mishra et al (22) (see details in the methods section), we consider a model, which hypothesises that SEP at each time point has an effect on the outcome, but does not restrict the effect sizes to be the same; (25-27) we call this a sensitive period model in line with previous analyses. (26) We also consider an 'adult accumulation' model which assumes no effect of childhood SEP once early adulthood and adult SEP are included in the model. An additional social mobility model allows the outcome to differ between those who remain in the less advantaged and more advantaged SEP category for all three time points (where the original model forced them to have the same mean outcome), and additional inter and intra generational models with fewer constraints are considered.

We used the structured approach to model selection to investigate which life course hypothesis best explained the relationship between life course SEP and adult crystallised ability in two socioeconomically contrasting studies from the UK; a nationally representative general population cohort (the MRC National Survey of Health and Development (NSHD)) and an occupational cohort of civil servants (the Whitehall II study). In both studies, life

For peer review only - http://bmjopen.bmj.com/site/about/guidelines.xhtml

course SEP has previously been related to crystallised cognitive ability, with accumulation models supported.(30, 31) The effect of inter-generational social mobility has also been supported in NSHD, with the socially mobile having cognitive levels intermediate to those in the two stable groups.(9) This finding is, however, consistent with the accumulation or sensitive period hypotheses where early- and late-life disadvantage have an additive effect, of equal or differing magnitudes. We directly compare multiple alternative models in order to identify the best fitting model and compare with previous findings, and evaluate the methodology used in such an approach. These findings can also be used to determine which life course models should be evaluated more thoroughly in future work. As less advantaged SEP and lower cognitive ability are major indicators of dropout in longitudinal studies,(32, 33) multiple imputation was carried out to account for missing data.

Methods

Study participants

The MRC National Survey of Health and Development (NSHD), also known as the 1946 British birth cohort study, is a stratified random sample of 5,362 children born in one week in March 1946. The 22nd wave of data collection on the full cohort took place in 2006-2011,(34) though no measure of crystallised ability was collected at this wave. By age 53, the age at which crystallised ability used in this study was tested, 8.7% of participants had died, 8.6% had emigrated and 2.2% were living abroad. Compared with national data, more men and women in the NSHD were employed full time at both ages 43 and 53,(35) but overall the NSHD was broadly representative.

For peer review only - http://bmjopen.bmj.com/site/about/guidelines.xhtml

The baseline survey of the Whitehall II study took place in 1985-1988, and recruited 10,308 non-industrial civil servants aged 35-55 (born 1930–1953).(36) Phase 11 was collected in 2012-13, with phases alternating between clinic visits and postal questionnaires. The cognitive outcome used in this study was collected at Phase 9 (2007-2009). By Phase 9 9.3% of the baseline respondents had died.

Measures

The National Adult Reading Test (NART) was administered at age 53 in NSHD, and the Mill Hill vocabulary test at Phase 9 (when participants were aged 58-74) in Whitehall II. The NART is a pronunciation test; participants are given a list of words which violate conventional grapheme-phoneme correspondence rules, and are therefore unlikely to be pronounced correctly unless the participant is familiar with the word.(37) The Mill Hill test (38) is a written vocabulary test which assesses the participants' understanding of words; participants must select the correct synonym for each word from a list of six alternatives. Both tests are well validated measures of verbal crystallised intelligence.(39, 40) To allow the results to be compared between the two studies, for each gender we standardised the NART and Mill Hill test scores.

SEP variables were dichotomised to carry out life course analyses. Childhood SEP in both studies was measured using father's occupational SEP, collected prospectively at age 4 in NSHD, and recalled retrospectively when participants were aged 44-69 in Whitehall II, categorized according to the Registrar General (RG) classification, dichotomised to manual/non-manual. Different markers of SEP in adulthood were used here, as Whitehall II is a sample of non-manual workers. Educational qualifications were the marker of early-adulthood SEP, collected until age 26 for NSHD and reported retrospectively for Whitehall II.

For peer review only - http://bmjopen.bmj.com/site/about/guidelines.xhtml

The less advantaged SEP groups were considered GCE 'O'-level (or equivalent) or below in NSHD and GCE 'A'-level/national diploma/certificate (or equivalent) or below in Whitehall II, due to the different distributions. In NSHD, adult SEP was own occupational SEP at age 43 (or age 36 if no occupation was recorded at 43), again dichotomised into manual/non-manual according to the Registrar General classification. In Whitehall II the last recorded civil service grade at phase 7 was used in the analyses, with Unified Grades 1-7 considered higher SEP. For participants no longer working in the civil service, the last recorded civil service grade was used.

Ethical approval for NSHD data collection at age 53 years was issued by North Thames Multi-centre Research Ethics Committee (MREC 98/1/121). Ethical approval for the Whitehall II study was obtained from the University College London Medical School Committee on the ethics of human research. All participants provided written informed consent.

Statistical analysis

All analyses were carried out using multiple imputation and repeated using complete cases. Since the data are not Missing Completely At Random (MCAR), multiple imputation results are presented as the primary analyses. The method of Mishra et al. compares a series of life course models with the saturated model to determine which model provides the best fit to the data.(22) The saturated model (Table 1) consists of the main effects for SEP at each of the three time points, the three two-way interactions and the single three-way interaction. This allows a different mean for each of the resulting eight possible life course SEP trajectories. Ordinary least squares multiple regression models were carried out.

For peer review only - http://bmjopen.bmj.com/site/about/guidelines.xhtml

All 12 other models considered here are nested within this saturated model, with explicit constraints on the parameters relating to different life course hypotheses. The model and constraints for each life course model tested are provided in Table 1.

For the accumulation model, the three binary SEP variables (0 for less advantaged SEP, 1 for more advantaged SEP) are summed to produce a score from 0-3. The three critical period models contain only the SEP measure at the relevant time point (childhood, early adulthood or midlife). Three types of social mobility were described by Mishra et al.:(22) inter-generational mobility, intra-generational social mobility, and any mobility.

The additional models we consider are as follows: the sensitive period model (25-27), in which each time point has an effect on the outcome, but the associations can differ in magnitude; an 'adult accumulation' model containing only early-adulthood and adult SEP; an inter generational social mobility model without constraints on the non-zero coefficients, an intra generational social mobility model without constraints on the non-zero coefficients, and an additional any mobility model containing a three-way interaction between SEP at each of the three time points, allowing the outcome to differ between those who remain in the less advantaged and more advantaged SEP for all three time points.

Each of the 12 life course models was tested against the saturated model using a partial F-test. If the result of a statistical test comparing two nested models is not statistically significant ($p>0.05$), there is no evidence the more complex model explains the data better than the simpler model; thus the simpler model is preferred. If multiple life course models were not significantly different to the saturated model, the model with the lowest Bayesian information criterion (BIC) was selected in complete case analyses. As the BIC cannot be applied with multiple imputation(41) we inspected the model coefficients and standard

For peer review only - http://bmjopen.bmj.com/site/about/guidelines.xhtml

deviations to select a model, by determining which model was best supported by the coefficients; for example when both the sensitive period model and adult accumulation model were not significantly different from the saturated model, we examined whether the childhood SEP variable was significant, and whether the coefficients of the two adult SEP variables were of similar magnitude.

As the Mill Hill test had been taken 0-5 times prior to phase 9 in the Whitehall II study, these analyses were adjusted for phase 9 age and the number of times the cognitive tests had previously been taken. There was a significant increase in Mill Hill test score with increasing number of times the test had been taken; a linear relationship between practice effects and crystallised cognitive function provided the best fit, with the lowest BIC (not shown). Analyses were carried out separately by gender due to evidence of gender interactions with level of educational qualification (p=0.002) and occupational SEP (p=0.04) in NSHD. Multiple imputation using chained equations was carried out using the Stata command *ice*. Separate imputation models were chosen for each gender in each dataset. The imputation model was chosen following the guidelines of van Buuren (42) and Carpenter and Plewis,(43) considering a wide selection of variables from all phases of the studies. Full details of the imputation models are provided in Appendix 1. Twenty imputations were used.(44) For the multiple imputation analyses, the crystallised cognitive ability measures were standardised after imputation, across all imputations.

We considered an alternative cutoff for SEP, dichotomising the variables into very low SEP vs. the rest as a sensitivity analysis, with approximately the least advantaged 10-20% considered as the very low SEP category (cutoffs: NSHD: father's occupational SEP: RG categories IV and V, educational qualifications: none attempted, occupational SEP: RG

For peer review only - http://bmjopen.bmj.com/site/about/guidelines.xhtml

category V. Whitehall II: father's occupational SEP: RG categories IV and V, educational qualifications: school matriculation or lower, occupational SEP: clerical/support jobs).

Analyses were carried out in Stata version 13.(45)

Results

In NSHD, adult crystallised ability at age 53 was available for 1,370 men and 1,455 women. Complete data on cognitive ability at age 53 and the three SEP variables were available for 1133 men (77% of those interviewed at age 53) and 1160 women (74%). Cognitive ability at Phase 9 of Whitehall II was available for 4,357 men and 1,687 women, of whom 2650 (56% of Phase 9 participants) and 900 (45%) also had complete data on the three SEP variables.

Table 2 gives the frequencies for each life course trajectory, for the complete case and multiple imputation analyses. For men the most common life course SEP trajectory was those who remained in the more advantaged category at all three time points (Table 2). For women, in NSHD the largest group was those whose father had a manual occupation, had a lower level of education, and had a non-manual occupation, whereas in Whitehall II the largest group were in the less advantaged category at all three time points. In both studies, a higher proportion of those with a missing outcome variable were in the more disadvantaged category (Table 3).

Life course analyses

In NSHD women and both genders in Whitehall II, in analyses using multiple imputation (Table 4), the sensitive period model fitted the data as well as the saturated model. In NSHD men, both the sensitive period model (p=0.98) and the adult accumulation model (p=0.31) fitted as well as the saturated model. Inspection of the coefficients from these models

For peer review only - http://bmjopen.bmj.com/site/about/guidelines.xhtml

suggested an association with childhood SEP, hence the sensitive period model was selected.

Within the supported sensitive period models, the results suggest that the association is stronger with adult SEP than childhood SEP. The models indicate that adult SEP had the largest coefficient for men in both studies and women in Whitehall II. For women in NSHD, early-adulthood SEP had the largest coefficient (Table 5). For men in NSHD the coefficients for early-adulthood (β=0.59 (95% CI:0.47-0.70)) and later adult SEP (β=0.65 (95% CI:0.53-0.76)) were very similar, and around 3 times the magnitude of the childhood SEP coefficient (β=0.21 (95% CI:0.10-0.32)). For women in Whitehall II, the childhood SEP coefficient was small (β=0.12 (95% CI:0.02-0.22)), with an intermediate effect of early-adulthood SEP (β=0.39 (95% CI:0.29-0.49)) compared to the effect of adult SEP (β=0.75 (95% CI:0.65-0.85)). The magnitude of the coefficients for Whitehall II men were similar to the coefficients for Whitehall II women (childhood SEP: β=0.15 (95% CI:0.09-0.21), early-adulthood SEP: β=0.37 (95% CI:0.31-0.43) and adult SEP: β=0.56 (95% CI:0.51-0.62)).

Complete case analyses

The sensitive period model remained the best fit for Whitehall II women and both genders in NSHD in agreement with the main analysis. However for Whitehall II men, all models, including the sensitive period model, were significantly poorer in fit than the saturated model (p=0.02) (supplementary table 1), therefore the saturated model where each trajectory has a different mean cognitive score is selected.

Sensitivity analysis

For peer review only - http://bmjopen.bmj.com/site/about/guidelines.xhtml

When SEP was dichotomised into very low SEP vs. the rest (supplementary table 2), for Whitehall II women the sensitive period model was no longer significantly different from the saturated model, however the intra generational model without constraints on the non-zero coefficients was not significantly different from the saturated model (p=0.0607). The same model was also no longer significantly different from the saturated model for Whitehall II men (p=0.4711), showing the importance of the interaction between educational qualifications and occupational SEP in Whitehall II. For NSHD women the inter generational social mobility model with no constraints on the non-zero effect sizes was not significantly different from the saturated model, demonstrating the importance of the interaction between childhood SEP and educational qualifications. For Whitehall II men and NSHD women, the model which provided the best fit to the data remained the same; inspection of the coefficients and backwards selection supported the sensitive period model, whereas for Whitehall II women, when using a cutoff of very low SEP, the intra generational social mobility model without restrictions on the non-zero coefficients provided the best fit to the data. In these women, the interaction term indicates an additional benefit of having at least O Levels and a non-clerical/support occupational grade (standardised β=0.40 (95% CI: 0.25-0.55)) over the additive effect.

Discussion

In NSHD and Whitehall II, for both genders our main analyses support sensitive period models suggesting more advantaged SEP at multiple points in the life course is associated with higher adult cognition. The results suggest the association with adult SEP is stronger than with childhood SEP. The consistency of these findings across the two cohorts despite differences in the measurement and distribution of adult SEP, emphasises the robustness of SEP at multiple stages of life in relation to crystallised cognitive ability, even within a limited

For peer review only - http://bmjopen.bmj.com/site/about/guidelines.xhtml

For peer review only

range of SEP. The NSHD and Whitehall II study sample very different populations, with Whitehall II a selective occupational cohort with all study members in a relatively advantaged non-manual occupational group in adulthood, and less variation in SEP across the life course than the population sample; everyone in Whitehall II would be in the more advantaged adult SEP category in the NSHD.

The general consistency in selection of the sensitive period model across the two studies emphasizes the importance of relative disadvantage throughout the life course, as well as absolute disadvantage, across the whole SEP distribution, though the intra generational social mobility model without constraints on the non-zero coefficients was supported in Whitehall II women when using a cutoff of very low SEP. Differences in the relative importance of SEP at different life stages were however observed between the two cohorts. The results were more similar between NSHD and Whitehall II in men than women. Educational qualifications were more important for women in the population-based cohort than the occupational cohort which is likely to be due to the different cut-points used as the majority of women in Whitehall II stay at school past the minimum school leaving age. It is likely to have impacted the type of future jobs available to women in the population cohort, with more highly educated women in NSHD likely to enter jobs which would increase their crystallised cognitive function, explaining the stronger relationship between education and crystallised cognitive function in this cohort. Occupational SEP in adulthood was more important for women in Whitehall II than NSHD; crystallized cognitive function accumulates through education and other experiences, including employment, with the type of tasks required in the civil service likely to contribute more than many other jobs held by women in a population cohort. Additionally, there is less variation in educational qualifications for

For peer review only - http://bmjopen.bmj.com/site/about/guidelines.xhtml

women in Whitehall II, increasing the relative importance of occupational SEP as a predictor of crystallised cognitive function.

Our findings using the structured modelling approach to consider various alternative models support previous work on these cohorts, which suggests the importance of SEP across each stage of life.(30, 31) This is also consistent with research supporting accumulation from other cohorts.(9, 46, 47) Previous support for an association with social mobility is consistent with accumulation and sensitive periods as well as social mobility defined here in terms of effect modification, though it is not easy to disentangle sensitive periods and social mobility. Those experiencing upwards or downwards mobility have sensitive period scores intermediate to those whose SEP is always low or always high (assuming all the sensitive periods have an effect in the same direction).(9, 46, 47) This highlights the advantage of using this methodology to directly compare the life course models, as if only one life course model had been considered, a sub-optimal model may have been selected. These results further demonstrate that crystallised ability can be augmented across the life course, consistent with its role as a marker of accumulating knowledge and experience. Interestingly, such augmentation can be observed with other verbal domains, such as memory (9, 30) and fluency,(9) although this is less pronounced for nonverbal function.(30) Using structural equation models, Richards and Sacker (30) and Singh-Manoux et al. (31) found that the effects of early life SEP were mediated through childhood cognition, education and adult occupation, with no direct effect remaining between childhood SEP and cognition, although direct effects were found for both education and adult occupation, suggesting an adult accumulation model. We observed a weaker association with childhood than adult SEP, supporting evidence that some of the association may be mediated via later

For peer review only - http://bmjopen.bmj.com/site/about/guidelines.xhtml

SEP, though this may also be due to methodological issues, such as childhood SEP being collected retrospectively in Whitehall II, and the dichotomisation of childhood SEP, compared to the categorisation and concurrent collection of adult SEP data. The structured approach does not test mediation models, so does not explicitly consider what proportion of the childhood SEP effect acts through later life SEP and whether any direct effect remains, though if the childhood SEP term in the sensitive period model is not significant, this implies no direct effect of childhood SEP remains.

We have used a relatively new structured modelling approach and allowed for missing data using multiple imputation. The main advantage of this life course methodology is its ability to compare multiple pre-specified life course models to a saturated model, testing whether each model is sufficiently complex to describe the relationship. This is an advantage over models which consider only one life course hypothesis. However, the method is based on P-values, hence the power to detect a difference in fit between models depends on the sample size, which may explain the selection of the saturated model for Whitehall II men in the complete case analyses. Further, it is not clear which model to select when more than one model fits the data as well as the saturated model. Due to the different number of parameters in the different life course models, it is not sufficient to compare P-values. We therefore used the BIC, which penalises the model for each additional parameter fitted; hence models with lower P-values were often selected as the best fit to the data. However, with large samples the BIC tends to favour simpler models due to the heavy penalty imposed for the number of parameters in the model. In the multiple imputation analyses, where the BIC cannot be used to compare the results, the simpler model was selected if it appeared reasonable in light of the coefficients in the relevant models.(27)

For peer review only - http://bmjopen.bmj.com/site/about/guidelines.xhtml

For peer review only

Multiple imputation was carried out, as the missing data were not MCAR. Although similar conclusions were reached for complete case and multiple imputation analyses, it is important that missing data are appropriately accounted for, as estimates can be biased when missing data are not MCAR yet complete case analyses are carried out. It is straightforward to apply multiple imputation to the structured modelling approach. However multiple imputation requires the assumption that data are MAR; the missing data is therefore a limitation of the study, but there is no reason to suspect that this would have a great impact on the findings.

The purpose of this paper was to describe the social inequalities in crystallised cognitive function. We cannot imply causality given the use of observational data and the assumptions on which the analyses are based. The selected hypothesis may subsequently be more precisely defined and studied further.(24) Unobserved confounders may explain some of the association between life course SEP and crystallised cognitive function in adulthood, for example genetic factors. There may also be modifiable mediators of the relationship, which are socially distributed, such as health related behaviours, which can subsequently be explored as possible interventions to reduce social inequalities in cognition. The fact that similar life course models including SEP at all three time periods were selected in two cohorts of differing socioeconomic backgrounds suggests that risk factors from across the life course are important in relation to cognition. We believe the results are likely to be reasonably generalizable, though note that the participants of both studies are predominantly white British and of a limited age range, so the results may not generalise to younger cohorts or other ethnic groups.

For peer review only - http://bmjopen.bmj.com/site/about/guidelines.xhtml

It was necessary to select three SEP variables to represent different life course stages and to dichotomise the SEP variables, resulting in the loss of information. Selecting the life course SEP variables was more of a challenge in the Whitehall II study; the adult SEP variable was the last recorded occupational SEP at phase 7, when the participants were aged 50-74. The ages at which the 3 selected SEP variables were measured were less evenly distributed for some participants than others, and the accumulation model corresponds less well to the length of time spent in more advantaged SEP conditions. What the different measures of SEP at the different stages in the life course represent must be considered: in this study childhood SEP is measured by father's occupational SEP, which is likely to be a proxy for material circumstances as well as the environment in the home, which will in turn influence level of education.(25) Higher educational qualifications provide human capital (skills, abilities and resources), a credential for selection into the labour market (48) as well as specific skills for work, and a sense of personal control, which is associated with health in general and healthy lifestyles, as well as improving work and economic conditions.(49)

In conclusion, the sensitive period hypothesis was supported when testing the relationship between life course SEP and cognitive ability in adulthood in these two contrasting cohorts, demonstrating the importance of each period of the life course, including childhood, though the relative effect sizes for each period varied by population and gender.

Acknowledgements

We thank the NSHD study members for their continuing support and members of the NSHD scientific and data collection teams, and all participating women and men in the Whitehall II Study, as well as all Whitehall II research scientists, study and data managers and clinical and administrative staff who make the study possible.

For peer review only - http://bmjopen.bmj.com/site/about/guidelines.xhtml

Data access

Whitehall II and NSHD data, protocols, and other metadata are available to bona fide researchers for research purposes. Please refer to the Whitehall II data sharing policy at http://www.ucl.ac.uk/whitehallII/data-sharing and the National Survey of Health and Development data sharing policy at http://www.nshd.mrc.ac.uk/data/

Contribution statement

Rebecca Landy co-designed the study and the analytic plan, analysed the data and drafted the manuscript. Jenny Head co-designed the study's analytic plan, and critically revised the manuscript. Marcus Richards helped focus and direct the study, and critically revised the literature review and Discussion section. Rebecca Hardy co-designed the study, co-designed the study's analytic plan, and critically revised the manuscript. All authors approve the submitted version of the manuscript. All authors declare that they have no competing interests.

Funding:

RL was supported by an ESRC PhD studentship at University College London. The UK Medical Research Council, British Heart Foundation, and the US National Institutes of Health (R01HL36310, R01AG013196) have supported collection of data in the Whitehall II Study. JH is partially supported by the Economic and Social Research Council (ES/K01336X/1). The MRC National Survey of Health and Development, MR and RH are funded by the UK Medical Research Council (MC_UU_12019/1, MC_UU_12019/2, MC_UU_12019/3).

For peer review only - http://bmjopen.bmj.com/site/about/guidelines.xhtml

Table 1: Model and constraints for each life course model tested

	Model specification	Constraints
Saturated model	$\alpha + \beta_1 S_1 + \beta_2 S_2 + \beta_3 S_3 + \theta_{12} S_1 S_2 + \theta_{23} S_2 S_3 + \theta_{13} S_1 S_3 + \theta_{123} S_1 S_2 S_3$	
No effect	α	
Accumulation models		
Accumulation	$\alpha + \beta_1 S_1 + \beta_2 S_2 + \beta_3 S_3$	$\beta_1 = \beta_2 = \beta_3$
Adult accumulation	$\alpha + \beta_2 S_2 + \beta_3 S_3$	$\beta_2 = \beta_3$
Sensitive period	$\alpha + \beta_1 S_1 + \beta_2 S_2 + \beta_3 S_3$	
Critical period models		
Childhood	$\alpha + \beta_1 S_1$	
Early adulthood	$\alpha + \beta_2 S_2$	
Adulthood	$\alpha + \beta_3 S_3$	
Social mobility models		
Inter generational	$\alpha + \beta_1 S_1 + \beta_2 S_2 + \beta_{12} S_1 S_2$	$\beta_1 + \beta_2 = -\beta_{12}$
Inter generational without constraints	$\alpha + \beta_1 S_1 + \beta_2 S_2 + \beta_{12} S_1 S_2$	
Intra generational	$\alpha + \beta_2 S_2 + \beta_3 S_3 + \beta_{23} S_2 S_3$	$\beta_2 + \beta_3 = -\beta_{23}$
Intra generational without constraints	$\alpha + \beta_2 S_2 + \beta_3 S_3 + \beta_{23} S_2 S_3$	
Any mobility	$\alpha + \beta_1 S_1 + \beta_2 S_2 + \beta_3 S_3 + \beta_{12} S_1 S_2 + \beta_{23} S_2 S_3$	$\beta_2 = \beta_1 + \beta_3 = -\beta_{12} = -\beta_{23}$
Any mobility with 3-way interaction	$\alpha + \beta_1 S_1 + \beta_2 S_2 + \beta_3 S_3 + \beta_{12} S_1 S_2 + \beta_{23} S_2 S_3 + \beta_{123} S_1 S_2 S_3$	$\beta_2 = \beta_1 + \beta_3 = -\beta_{12} = -\beta_{23}$

S_i are the binary life course SEP variables, where S_i is equal to 0 when the participant is in the less advantaged SEP group at time point *i*, and 1 when the participant is in the more advantaged SEP group at time point *i*. The saturated model consists of the main effects for SEP at each of the three time points, as well as the three two-way interactions and the single three-way interaction.

For peer review only - http://bmjopen.bmj.com/site/about/guidelines.xhtml

Table 2: SEP trajectory frequencies, using father's occupational SEP (childhood SEP), educational qualifications (early-adulthood SEP) and own adult occupational SEP (adult SEP)

			NSHD								Whitehall II							
			Men				Women				Men				Women			
			Complete case		Multiple imputation		Complete case		Multiple imputation		Complete case		Multiple imputation		Complete case		Multiple imputation	
Childhood SEP	Early-adulthood SEP	Adult SEP	N	%	N	%	N	%	N	%	N	%	N	%	N	%	N	%
0	0	0	263	23	789.8	29	226	19	565	22	454	17	1382.2	20	341	38	1510.9	44
0	0	1	175	15	476.6	17	327	28	731	29	281	11	652.4	9	25	3	67.2	2
0	1	0	55	5	98.2	4	4	0	13.8	1	87	3	235.8	3	19	2	83.4	2
0	1	1	151	13	323.6	12	90	8	172.7	7	236	9	555.2	8	24	3	58.8	2
1	0	0	62	5	181.1	4	59	5	153.7	6	499	19	1434.2	21	240	27	1005.7	29
1	0	1	111	10	295.5	11	203	18	449.5	18	367	14	849.1	12	52	6	131.8	4
1	1	0	24	2	59	2	11	1	23.1	1	165	6	489.9	7	83	9	268.1	8
1	1	1	292	26	591.3	21	240	21	438.3	17	561	21	1296.4	19	116	13	287.4	8
Total			1133	100	2815	100	1160	100	2547	100	2650	100	6895	100	900	100	3413	100

* Mean of 20 imputations. SEP=0 if the participant is in the less advantaged SEP category, and SEP=1 if the participant is in the more advantaged SEP category.

For peer review only - http://bmjopen.bmj.com/site/about/guidelines.xhtml

Table 3: Distribution of childhood SEP (S_1) and early- adulthood SEP (S_2) for men and women in each of the NSHD and Whitehall II study by whether the outcome variable was observed

		Men				Women			
		Observed crystallised ability		Missing crystallised ability		Observed crystallised ability		Missing crystallised ability	
Whitehall II		N	%	N	%	N	%	N	%
S_1	0	1189	40	792	45	516	47	619	56
	1	1814	60	979	55	591	53	480	44
S_2	0	2273	59	637	65	974	71	443	81
	1	1569	41	344	35	398	29	102	19
NSHD									
S_1	0	720	57	680	62	749	56	517	64
	1	543	43	412	38	583	44	296	36
S_2	0	719	55	695	69	974	71	593	80
	1	582	45	312	31	405	29	152	20

For peer review only

For peer review only - http://bmjopen.bmj.com/site/about/guidelines.xhtml

Table 4: Results of tests comparing alternative life course hypotheses for crystallised cognitive ability (NSHD: NART score, Whitehall II: Mill Hill test score) with the saturated model (NSHD models are unadjusted, Whitehall II models are adjusted for age and number of times the Mill Hill test has previously been taken. Multiple imputation is implemented to account for missing data)

	NSHD				Whitehall II			
	Women (N=2,547)		Men (N=2,815)		Women (N=3,413)		Men (N=6,895)	
**Hypothesis**	F statistic	P-value*	F statistic	P-value*	F statistic	P-value*	F statistic	P-value*
No effect	94.00	<0.0001	86.99	<0.0001	58.98	<0.0001	129.45	<0.0001
Accumulation models								
Accumulation	6.11	<0.0001	7.05	<0.0001	13.44	<0.0001	18.03	<0.0001
Adult accumulation	5.64	<0.0001	1.19	**0.3116**	4.28	0.0003	7.89	<0.0001
Sensitive period	1.39	**0.2375**	0.10	**0.9821**	0.97	**0.4224**	0.32	**0.8670**
Critical period models								
Childhood SEP	63.58	<0.0001	77.04	<0.0001	61.78	<0.0001	138.57	<0.0001
Early-adulthood SEP	19.95	<0.0001	32.40	<0.0001	30.50	<0.0001	72.73	<0.0001
Adult SEP	61.93	<0.0001	32.94	<0.0001	12.21	<0.0001	34.71	<0.0001
Social mobility models								
Inter generational	117.88	<0.0001	111.71	<0.0001	82.01	<0.0001	171.74	<0.0001
Inter generational without constraints	15.23	<0.0001	38.33	<0.0001	44.99	<0.0001	102.97	<0.0001
Intra generational	117.00	<0.0001	123.12	<0.0001	64.91	<0.0001	174.27	<0.0001
Intra generational without constraints	10.84	<0.0001	4.77	0.0009	2.81	0.0257	8.08	<0.0001
Any mobility	128.51	<0.0001	112.79	<0.0001	69.45	<0.0001	152.22	<0.0001
Any mobility with 3-way interaction	31.25	<0.0001	25.20	<0.0001	9.28	<0.0001	28.91	<0.0001

* The P-values test whether the life course model is significantly different from the saturated model. P-values in bold indicate where a model fits as well as the saturated model

For peer review only

For peer review only - http://bmjopen.bmj.com/site/about/guidelines.xhtml

Table 5: Standardised coefficients for each term in the relaxed accumulation models, in the NSHD and Whitehall II, by gender.

	Men				Women			
	Complete Case		Multiple imputation		Complete Case		Multiple imputation	
	Coeff	95% CI	Coeff	95% CI	Coeff	95% CI	Coeff	95% CI
	Unadjusted NSHD				Unadjusted NSHD			
Childhood SEP	0.23	(0.11, 0.35)	0.21	(0.10, 0.32)	0.32	(0.19, 0.44)	0.36	(0.25, 0.48)
Early-adulthood SEP	0.51	(0.38, 0.64)	0.59	(0.47, 0.70)	0.83	(0.70, 0.96)	0.82	(0.71, 0.93)
Adult SEP	0.66	(0.52, 0.80)	0.65	(0.53, 0.76)	0.51	(0.36, 0.65)	0.50	(0.36, 0.63)
Constant	-0.74	(-0.85, -0.64)	-0.70	(-0.79, -0.60)	-0.79	(-0.91, -0.67)	-0.72	(-0.82, -0.62)
	Whitehall II*				Whitehall II			
Childhood SEP			0.15	(0.09, 0.21)	0.14	(0.02, 0.26)	0.12	(0.02, 0.22)
Early-adulthood SEP			0.37	(0.31, 0.43)	0.43	(0.28, 0.57)	0.39	(0.29, 0.49)
Adult SEP			0.56	(0.51, 0.62)	0.62	(0.47, 0.77)	0.75	(0.65, 0.85)
Age			0.00	(-0.01, 0.00)	-0.01	(-0.02, -0.00)	-0.02	(-0.02, -0.01)
Number of times previously taken cognitive tests			0.07	(0.03, 0.10)	0.06	(-0.01, 0.14)	0.13	(0.09, 0.18)
Constant			-0.44	(-0.73, -0.14)	0.38	(-0.30, 1.05)	0.68	(0.22, 1.14)

* None of the life course models considered fit the data as well as the saturated model in the complete case analysis of men in Whitehall II.

For peer review only - http://bmjopen.bmj.com/site/about/guidelines.xhtml

1. Cattell RB. Abilities: Their structure, growth, and action. New York: Houghton Mifflin; 1971.
2. Carroll JB. Human cognitive abilities: a survey of factor analytic studies. Cambridge UK: Cambridge University Press; 1993.
3. Deary IJ, Batty GD. Cognitive epidemiology. J Epidemiol Community Health. 2007;61(5):378-84.
4. B. Bosworth KWS, Hayden. Survival effects in cognitive function, cognitive style, and sociodemographic variables in the Seattle Longitudinal Study. Experimental Aging Research. 1999;25(2):121-39.
5. Lubinski D. Cognitive epidemiology: With emphasis on untangling cognitive ability and socioeconomic status. Intelligence. 2009;37(6):625-33.
6. Singh-Manoux A. Commentary: Is it time to redefine cognitive epidemiology? International journal of epidemiology. 2010:dyq123.
7. Luo Y, Waite LJ. The impact of childhood and adult SES on physical, mental, and cognitive well-being in later life. Journals of Gerontology Series B-Psychological Sciences and Social Sciences. 2005;60(2):S93-S101.
8. Turrell G, Lynch JW, Kaplan GA, Everson SA, Helkala EL, Kauhanen J, et al. Socioeconomic position across the lifecourse and cognitive function in late middle age. J Gerontol B Psychol Sci Soc Sci. 2002;57(1):S43-51.
9. Hatch SL, Feinstein L, Link BG, Wadsworth MEJ, RichardS M. The continuing benefits of education: Adult education and midlife cognitive ability in the British 1946 birth cohort. Journals of Gerontology Series B-Psychological Sciences and Social Sciences. 2007;62(6):S404-S14.
10. Lee S, Kawachi I, Berkman LF, Grodstein F. Education, other socioeconomic indicators, and cognitive function. Am J Epidemiol. 2003;157(8):712-20.
11. Kaplan GA, Turrell G, Lynch JW, Everson SA, Helkala E-L, Salonen JT. Childhood socioeconomic position and cognitive function in adulthood. International Journal of Epidemiology. 2001;30(2):256-63.
12. Everson-Rose SA, de Leon CFM, Bienias JL, Wilson RS, Evans DA. Early life conditions and cognitive functioning in later life. American Journal of Epidemiology. 2003;158(11):1083-9.
13. Packard CJ, Bezlyak V, McLean JS, Batty GD, Ford I, Burns H, et al. Early life socioeconomic adversity is associated in adult life with chronic inflammation, carotid atherosclerosis, poorer lung function and decreased cognitive performance: a cross-sectional, population-based study. BMC public health. 2011;11(1):42.
14. Maurer J. Height, education and later-life cognition in Latin America and the Caribbean. Economics & Human Biology. 2010;8(2):168-76.
15. Gallacher JE, Elwood PC, Hopkinson C, Rabbitt PM, Stollery BT, Sweetnam PM, et al. Cognitive function in the Caerphilly study: associations with age, social class, education and mood. European journal of epidemiology. 1999;15(2):161-9.
16. Zhao JH, Brunner E, Kumari M, Singh-Manoux A, Hawe E, Talmud P, et al. APOE polymorphism, socioeconomic status and cognitive function in mid-life. Social psychiatry and psychiatric epidemiology. 2005;40(7):557-63.
17. Jorm AF, Rodgers B, Henderson AS, Korten AE, Jacomb PA, Christensen H, et al. Occupation type as a predictor of cognitive decline and dementia in old age. Age and ageing. 1998;27(4):477-83.
18. Tomalski P, Johnson MH. The effects of early adversity on the adult and developing brain. Current opinion in psychiatry. 2010;23(3):233-8.
19. Ben-Shlomo Y, Kuh D. A life course approach to chronic disease epidemiology: conceptual models, empirical challenges and interdisciplinary perspectives. International journal of epidemiology. 2002;31(2):285-93.
20. Ben-Shlomo Y, Cooper R, Kuh D. The last two decades of life course epidemiology, and its relevance for research on ageing. International Journal of Epidemiology. 2016;45(4):973-88.
21. Hallqvist J, Lynch J, Bartley M, Lang T, Blane D. Can we disentangle life course processes of accumulation, critical period and social mobility? An analysis of disadvantaged socio-economic

For peer review only - http://bmjopen.bmj.com/site/about/guidelines.xhtml

positions and myocardial infarction in the Stockholm Heart Epidemiology Program. Social science & medicine. 2004;58(8):1555-62.
22. Mishra G, Nitsch D, Black S, De Stavola B, Kuh D, Hardy R. A structured approach to modelling the effects of binary exposure variables over the life course. International journal of epidemiology. 2009;38(2):528-37.
23. Howe LD, Chaturvedi N, Lawlor DA, Ferreira DL, Fraser A, Smith GD, et al. Rapid increases in infant adiposity and overweight/obesity in childhood are associated with higher central and brachial blood pressure in early adulthood. Journal of hypertension. 2014;32(9):1789.
24. Smith AD, Heron J, Mishra G, Gilthorpe MS, Ben-Shlomo Y, Tilling K. Model selection of the effect of binary exposures over the life course. Epidemiology. 2015;26(5):719-26.
25. Robertson T, Popham F, Benzeval M. Socioeconomic position across the lifecourse & allostatic load: data from the West of Scotland Twenty-07 cohort study. BMC public health. 2014;14(1):184.
26. Mishra GD, Chiesa F, Goodman A, De Stavola B, Koupil I. Socio-economic position over the life course and all-cause, and circulatory diseases mortality at age 50-87 years: results from a Swedish birth cohort. European journal of epidemiology. 2013;28(2):139-47.
27. Murray ET, Mishra GD, Kuh D, Guralnik J, Black S, Hardy R. Life course models of socioeconomic position and cardiovascular risk factors: 1946 birth cohort. Annals of Epidemiology. 2011;21(8):589-97.
28. Jones R, Hardy R, Sattar N, Deanfield JE, Hughes A, Kuh D, et al. Novel coronary heart disease risk factors at 60-64 years and life course socioeconomic position: The 1946 British birth cohort. Atherosclerosis. 2015;238(1):70.
29. Ben-Shlomo Y, Mishra G, Kuh D. Life course epidemiology. In: Ahrens W, Pigeot I, editors. Handbook of Epidemiology second ed: Springer; 2013.
30. Richards M, Sacker A. Lifetime antecedents of cognitive reserve. Journal of Clinical and Experimental Neuropsychology. 2003;25(5):614-24.
31. Singh-Manoux A, Richards M, Marmot M. Socioeconomic position across the lifecourse: How does it relate to cognitive function in mid-life? Annals of Epidemiology. 2005;15(8):572-8.
32. Richards M, Shipley B, Fuhrer R, Wadsworth ME. Cognitive ability in childhood and cognitive decline in mid-life: longitudinal birth cohort study. Bmj. 2004;328(7439):552.
33. Matthews FE, Chatfield M, Freeman C, McCracken C, Brayne C, CFAS M. Attrition and bias in the MRC cognitive function and ageing study: an epidemiological investigation. BMC public health. 2004;4(1):12.
34. Kuh D, Pierce M, Adams J, Deanfield J, Ekelund U, Friberg P, et al. Cohort profile: updating the cohort profile for the MRC National Survey of Health and Development: a new clinic-based data collection for ageing research. International journal of epidemiology. 2011;40(1):e1-e9.
35. Wadsworth M, Butterworth S, Hardy R, Kuh D, Richards M, Langenberg C, et al. The life course prospective design: an example of benefits and problems associated with study longevity. Social science & medicine. 2003;57(11):2193-205.
36. Ferrie JE, Kivimaki M, Singh-Manoux A, Shortt A, Martikainen P, Head J, et al. Non-response to baseline, non-response to follow-up and mortality in the Whitehall II cohort. International journal of epidemiology. 2009;38(3):831-7.
37. Nelson HE, Willison JR. National Adult Reading Test (NART) (2nd ed.): Windsor: NFER Nelson; 1991.
38. Raven J. Guide to Using the Mill Hill Vocabulary Scale with the Progressive Matrices Scales. Oxford, England: H.K. Lewis & Co.; 1958.
39. Crawford JR, Deary IJ, Starr J, Whalley LJ. The NART as an index of prior intellectual functioning: a retrospective validity study covering a 66-year interval. Psychological medicine. 2001;31(03):451-8.
40. Franzen M.D. Reliability and Validity in Neuropsychological Assessment. second ed: Springer Science & Business Media; 2013.

For peer review only - http://bmjopen.bmj.com/site/about/guidelines.xhtml

41. Statacorp. Stata help file for mim command. 2011.
42. Van Buuren S, Boshuizen HC, Knook DL. Multiple imputation of missing blood pressure covariates in survival analysis. Statistics in medicine. 1999;18(6):681-94.
43. Carpenter J, Plewis I. Analysing Longitudinal Studies with Non-response: Issues and statistical methods. In: Willians M, Vogt W, editors. The SAGE Handbook of Innovation in Social Research Methods: SAGE Publications Ltd; 2011.
44. Sterne JA, White IR, Carlin JB, Spratt M, Royston P, Kenward MG, et al. Multiple imputation for missing data in epidemiological and clinical research: potential and pitfalls. Bmj. 2009;338.
45. Statacorp. Stata Statistical Software: Release 11. College Station, TX: Statacorp LP; 2009.
46. Turrell G, Lynch JW, Kaplan GA, Everson SA, Helkala E-L, Kauhanen J, et al. Socioeconomic position across the lifecourse and cognitive function in late middle age. The Journals of Gerontology Series B: Psychological Sciences and Social Sciences. 2002;57(1):S43-S51.
47. Luo Y, Waite LJ. The impact of childhood and adult SES on physical, mental, and cognitive well-being in later life. The Journals of Gerontology Series B: Psychological Sciences and Social Sciences. 2005;60(2):S93-S101.
48. Collins R. The credential society: An historical sociology of education and stratification. New York, NY: Academic Press; 1979.
49. Ross CE, Mirowsky J. Refining the association between education and health: The effects of quantity, credential, and selectivity. Demography. 1999;36(4):445-60.

For peer review only

For peer review only - http://bmjopen.bmj.com/site/about/guidelines.xhtml

Appendix 1

Multiple imputation was carried out using the Stata command *ice*, created by Patrick Royston (118;119), which samples from the posterior predictive distribution $p(Y_{missing}|Y_{observed}, X)$. For 20 imputations, 20 independent selections are made from the posterior predictive distribution. All the variables which are thought to predict or be associated with the missing values should be included in the model used to form the posterior predictive distribution, the imputation model. When choosing which variables to include in the imputation model, it is important to also include any variables that may be important in subsequent analyses, including the variable(s) with missing data and the outcome of interest. It has been shown that including as many explanatory variables in the imputation model as possible makes the MAR assumption more plausible (van Buuren et al., 1999). Although including redundant predictors may be expected to reduce the precision of the final estimates, Kenward and Carpenter (Kenward and Carpenter, 2007) note that this effect is typically not large, concluding that it is better to err on the side of including too many variables in the imputation model rather than too few, as excluding an important predictor of missingness could cause bias.

van Buuren (van Buuren et al., 1999) and Carpenter and Plewis (Carpenter and Plewis, 2011) suggest similar methods for selecting the variables to include in the imputation model from a large database:

1. Include all variables that appear in the complete-data model of interest.
2. Include the variables which were predictive of missing data.
3. Check whether the variables included in step 2 were associated with the variables in the model of interest.
4. Auxiliary variables can also be included to make the assumption of MAR more likely. Auxiliary variables are predictive of any of the variables in the imputation model.

Binary variables were imputed using logistic regression, ordered categorical variables using ordered logistic regression, and unordered categorical variables using multinomial regression.

For peer review only

Variables included in the imputation model for Whitehall II men, in addition to those in the model of interest

Phase of data collection	Variables
1	Year of birth, longstanding illness, current cigarette smoker, age when finished full time education, isolation scale score, usually pressed for time, believe no one cares much about you, state of health in the last year, accommodation type, mother's age when finished education
3	Job involves travel away from home, marital status, last recorded civil service grade, memory score, AH4 score, verbal fluency (S-words, animals), Mill Hill score
4	Ever told had anxiety, ever told had depression,
5	Childhood emotional deprivation, childhood material deprivation, ever told high blood pressure, deprivation score, feel might as well give up, financial security in the next 10 years, last recorded civil service grade, memory score, AH4 score, verbal fluency (S-words, animals), Mill Hill score
7	Clinic or home visit, still working as a civil servant, general health, CASP total score, my health stops me doing things I want to do, MMSE total score, memory score, AH4 score, verbal fluency (S-words, animals), Mill Hill score
9	General health, difficulty paying bills, MMSE total score, marital status, last recorded civil service grade, memory score, AH4 score, verbal fluency (S-words, animals)

For peer review only

Variables included in the imputation model for Whitehall II women, in addition to those in the model of interest

Phase of data collection	Variables
1	Year of birth, longstanding illness, current cigarette smoker, age when finished full time education, isolation scale score, usually pressed for time, state of health in the last year, accommodation type, mother's age when finished education, satisfied by job as a whole, civil service grade
3	Job involves travel away from home, marital status, last recorded civil service grade, memory score, AH4 score, verbal fluency (S-words, animals), Mill Hill score
4	Ever told had depression
5	Childhood emotional deprivation, childhood material deprivation, ever told high blood pressure, feel might as well give up, last recorded civil service grade, memory score, AH4 score, verbal fluency (S-words, animals), Mill Hill score
7	Clinic or home visit, still working as a civil servant, general health, my health stops me doing things I want to do, MMSE total score, memory score, AH4 score, verbal fluency (S-words, animals), Mill Hill score
9	General health, difficulty paying bills, MMSE total score, marital status, last recorded civil service grade, memory score, AH4 score, verbal fluency (S-words, animals)

For peer review only

Variables included in the imputation model for NSHD men, in addition to those in the model of interest

Participant's Age	Variables
0	Father's occupational SEP
2	Childhood household amenities
4	Child has own bed, clothes repair, yard or garden, child's shoes, cleanliness of house, cleanliness of child, mother's management and understanding of the child, crowding, repair of dwelling
8	Reading score, sentence completion score, picture intelligence score, vocabulary score
10	Difficult to discipline
11	Father's occupational SEP, non-verbal reasoning score, reading score, verbal reasoning score, arithmetic score, vocabulary score, household amenities
13	Have to stick up for myself
15	Non-verbal reasoning score, reading score, verbal reasoning score, mathematics score, truancy during the past year
26	Own occupational SEP, happiness, job stress, brought up in a faith, currently have a religion
36	Marital status, current smoking status, ever been unemployed, own occupational SEP, ever had cancer, does religious upbringing effect life now
43	How many friends or relatives could you visit at any time without waiting for an invite, verbal memory score
53	Verbal fluency score

For peer review only

Variables included in the imputation model for NSHD women, in addition to those in the model of interest

Participant's Age	Variables
0	Father's occupational SEP
2	Childhood household amenities
4	Child has own bed, clothes repair, yard or garden, child's shoes, cleanliness of house, cleanliness of child, mother's management and understanding of the child, crowding, repair of dwelling, age of dwelling
8	Reading score, sentence completion score, picture intelligence score, vocabulary score
10	Difficult to discipline
11	Father's occupational SEP, non-verbal reasoning score, reading score, verbal reasoning score, arithmetic score, vocabulary score, household amenities
13	Have to stick up for myself
15	Non-verbal reasoning score, reading score, verbal reasoning score, mathematics score, truancy during the past year
26	Own occupational SEP, happiness, job stress, brought up in a faith, currently have a religion
31	Hospital admissions
36	Marital status, current smoking status, ever been unemployed, own occupational SEP, ever had cancer, does religious upbringing effect life now, blood pressure, how confident feel in myself
43	Head of household SEP, verbal memory score
53	Verbal fluency score

References:

CARPENTER, J. & PLEWIS, I. (2011) Analysing Longitudinal Studies with Non-response: Issues and statistical methods. IN WILLIANS, M. & VOGT, W. (Eds.) *The SAGE Handbook of Innovation in Social Research Methods.* SAGE Publications Ltd.

KENWARD, M. G. & CARPENTER, J. (2007) Multiple imputation: current perspectives. *Statistical Methods in Medical Research,* 16**,** 199-218.

VAN BUUREN, S., BOSHUIZEN, H. C. & KNOOK, D. L. (1999) Multiple imputation of missing blood pressure covariates in survival analysis. *Statistics in medicine,* 18**,** 681-694.

For peer review only

Supplementary Table 1: Complete case results of tests comparing alternative life course hypotheses for crystallised cognitive ability (NSHD: NART score, Whitehall II: Mill Hill test score) with the saturated model (NSHD models are unadjusted, Whitehall II models are adjusted for age and number of times the Mill Hill test has previously been taken.

	NSHD				Whitehall II			
	Women		Men		Women		Men	
**Hypothesis**	F statistic	P-value*	F statistic	P-value*	F statistic	P-value*	F statistic	P-value*
No effect	80.72	<0.0001	53.99	<0.0001	31.16	<0.0001	70.33	<0.0001
Accumulation models								
Accumulation	4.90	0.0001	4.37	0.0002	4.74	0.0001	12.32	<0.0001
Adult accumulation	7.18	<0.0001	3.24	0.0037	1.85	**0.0861**	5.73	<0.0001
Sensitive period	2.02	**0.0891**	0.69	**0.5967**	0.93	**0.4459**	2.80	0.0248
Critical period models								
Childhood	51.80	<0.0001	45.41	<0.0001	28.49	<0.0001	74.81	<0.0001
Early adulthood	23.43	<0.0001	21.63	<0.0001	13.89	<0.0001	40.90	<0.0001
Adulthood	52.01	<0.0001	21.11	<0.0001	8.55	<0.0001	19.16	<0.0001
Social mobility models								
Inter generational	109.03	<0.0001	70.95	<0.0001	40.48	<0.0001	87.81	<0.0001
Inter generational without constraints	21.55	<0.0001	24.66	<0.0001	17.74	<0.0001	55.35	<0.0001
Intra generational	89.89	<0.0001	70.08	<0.0001	40.78	<0.0001	95.64	<0.0001
Intra generational without constraints	6.65	<0.0001	4.52	0.0013	2.23	**0.0639**	6.20	0.0001
Any mobility	102.18	<0.0001	71.89	<0.0001	38.22	<0.0001	79.28	<0.0001
Any mobility with 3-way interaction	29.44	<0.0001	20.84	<0.0001	5.45	0.0002	8.90	<0.0001

* The P-values test whether the life course model is significantly different from the saturated model. P-values in bold indicate where a model fits as well as the saturated model.

For peer review

Supplementary Table 2: Results of tests comparing alternative life course hypotheses for crystallised cognitive ability (NSHD: NART score, Whitehall II: Mill Hill test score) with the saturated model, using a cutoff of very low SEP vs. the rest. NSHD models are unadjusted, Whitehall II models are adjusted for age and number of times the Mill Hill test has previously been taken. Multiple imputation is implemented to account for missing data

	NSHD				Whitehall II			
	Women (N=2,547)		Men (N=2,815)		Women (N=3,413)		Men (N=6,895)	
**Hypothesis**	F statistic	P-value*	F statistic	P-value*	F statistic	P-value*	F statistic	P-value*
No effect	72.65	<0.0001	58.47	<0.0001	103.75	<0.0001	72.88	<0.0001
Accumulation models								
Accumulation	17.97	<0.0001	15.55	<0.0001	24.03	<0.0001	18.8	<0.0001
Adult accumulation	5.97	<0.0001	0.57	**0.7258**	11.80	<0.0001	6.39	<0.0001
Sensitive period	0.38	**0.8216**	0.24	**0.9166**	6.30	0.0001	0.35	**0.8413**
Critical period models								
Childhood SEP	68.58	<0.0001	61.92	<0.0001	115.86	<0.0001	78.89	<0.0001
Early-adulthood SEP	3.36	0.0029	4.42	0.0002	73.79	<0.0001	49.68	<0.0001
Adult SEP	75.73	<0.0001	63.20	<0.0001	17.16	<0.0001	21.68	<0.0001
Social mobility models								
Inter generational	64.81	<0.0001	48.99	<0.0001	113.90	<0.0001	71.37	<0.0001
Inter generational without constraints	1.63	**0.1663**	3.66	0.0060	106.95	<0.0001	69.22	<0.0001
Intra generational	31.16	<0.0001	14.77	<0.0001	119.40	<0.0001	55.48	<0.0001
Intra generational without constraints	3.45	0.0085	2.92	0.0207	0.89	**0.4711**	2.28	**0.0607**
Any mobility	44.54	<0.0001	29.66	<0.0001	58.06	<0.0001	35.08	<0.0001
Any mobility with 3-way interaction	29.37	<0.0001	24.99	<0.0001	8.50	<0.0001	10.58	<0.0001

* The P-values test whether the life course model is significantly different from the saturated model. P-values in bold indicate where a model fits as well as the saturated model.

For peer review only

STROBE Statement—Checklist of items that should be included in reports of ***cohort studies***

	Item No	Recommendation	Page
**Title and abstract**	1	(*a*) Indicate the study's design with a commonly used term in the title or the abstract	1
		(*b*) Provide in the abstract an informative and balanced summary of what was done and what was found	2
**Introduction**			
Background/rationale	2	Explain the scientific background and rationale for the investigation being reported	5-6
Objectives	3	State specific objectives, including any prespecified hypotheses	6-7
**Methods**			
Study design	4	Present key elements of study design early in the paper	7-8
Setting	5	Describe the setting, locations, and relevant dates, including periods of recruitment, exposure, follow-up, and data collection	7-8
Participants	6	(*a*) Give the eligibility criteria, and the sources and methods of selection of participants. Describe methods of follow-up	7-8
		(*b*) For matched studies, give matching criteria and number of exposed and unexposed	n/a
Variables	7	Clearly define all outcomes, exposures, predictors, potential confounders, and effect modifiers. Give diagnostic criteria, if applicable	8-9, 11
Data sources/ measurement	8*	For each variable of interest, give sources of data and details of methods of assessment (measurement). Describe comparability of assessment methods if there is more than one group	8-9
Bias	9	Describe any efforts to address potential sources of bias	9
Study size	10	Explain how the study size was arrived at	7-8
Quantitative variables	11	Explain how quantitative variables were handled in the analyses. If applicable, describe which groupings were chosen and why	8-9
Statistical methods	12	(*a*) Describe all statistical methods, including those used to control for confounding	9-11
		(*b*) Describe any methods used to examine subgroups and interactions	n/a
		(*c*) Explain how missing data were addressed	9
		(*d*) If applicable, explain how loss to follow-up was addressed	9
		(*e*) Describe any sensitivity analyses	9, 11
**Results**			
Participants	13*	(a) Report numbers of individuals at each stage of study—eg numbers potentially eligible, examined for eligibility, confirmed eligible, included in the study, completing follow-up, and analysed	7-8
		(b) Give reasons for non-participation at each stage	7-8
		(c) Consider use of a flow diagram	n/a
Descriptive data	14*	(a) Give characteristics of study participants (eg demographic, clinical, social) and information on exposures and potential confounders	12
		(b) Indicate number of participants with missing data for each variable of interest	22
		(c) Summarise follow-up time (eg, average and total amount)	7-8
Outcome data	15*	Report numbers of outcome events or summary measures over time	12

For peer review only

For peer review only - http://bmjopen.bmj.com/site/about/guidelines.xhtml

Main results	16	(*a*) Give unadjusted estimates and, if applicable, confounder-adjusted estimates and their precision (eg, 95% confidence interval). Make clear which confounders were adjusted for and why they were included	13, 25
		(*b*) Report category boundaries when continuous variables were categorized	8-9
		(*c*) If relevant, consider translating estimates of relative risk into absolute risk for a meaningful time period	n/a
Other analyses	17	Report other analyses done—eg analyses of subgroups and interactions, and sensitivity analyses	12-13
**Discussion**			
Key results	18	Summarise key results with reference to study objectives	14-15
Limitations	19	Discuss limitations of the study, taking into account sources of potential bias or imprecision. Discuss both direction and magnitude of any potential bias	18-19
Interpretation	20	Give a cautious overall interpretation of results considering objectives, limitations, multiplicity of analyses, results from similar studies, and other relevant evidence	19
Generalisability	21	Discuss the generalisability (external validity) of the study results	18
**Other information**			
Funding	22	Give the source of funding and the role of the funders for the present study and, if applicable, for the original study on which the present article is based	20

*Give information separately for exposed and unexposed groups.

Note: An Explanation and Elaboration article discusses each checklist item and gives methodological background and published examples of transparent reporting. The STROBE checklist is best used in conjunction with this article (freely available on the Web sites of PLoS Medicine at http://www.plosmedicine.org/, Annals of Internal Medicine at http://www.annals.org/, and Epidemiology at http://www.epidem.com/). Information on the STROBE Initiative is available at http://www.strobe-statement.org.

For peer review only

For peer review only - http://bmjopen.bmj.com/site/about/guidelines.xhtml
